# Supplementary figures and images for: Double-blinded randomized controlled trial to reveal the effects of Brazilian propolis intake on rheumatoid arthritis disease activity index; BeeDAI
Source: PLoS One. 2021 May 27;16(5):e0252357. doi: 10.1371/journal.pone.0252357 (PMC8158978; doi:10.1371/journal.pone.0252357)

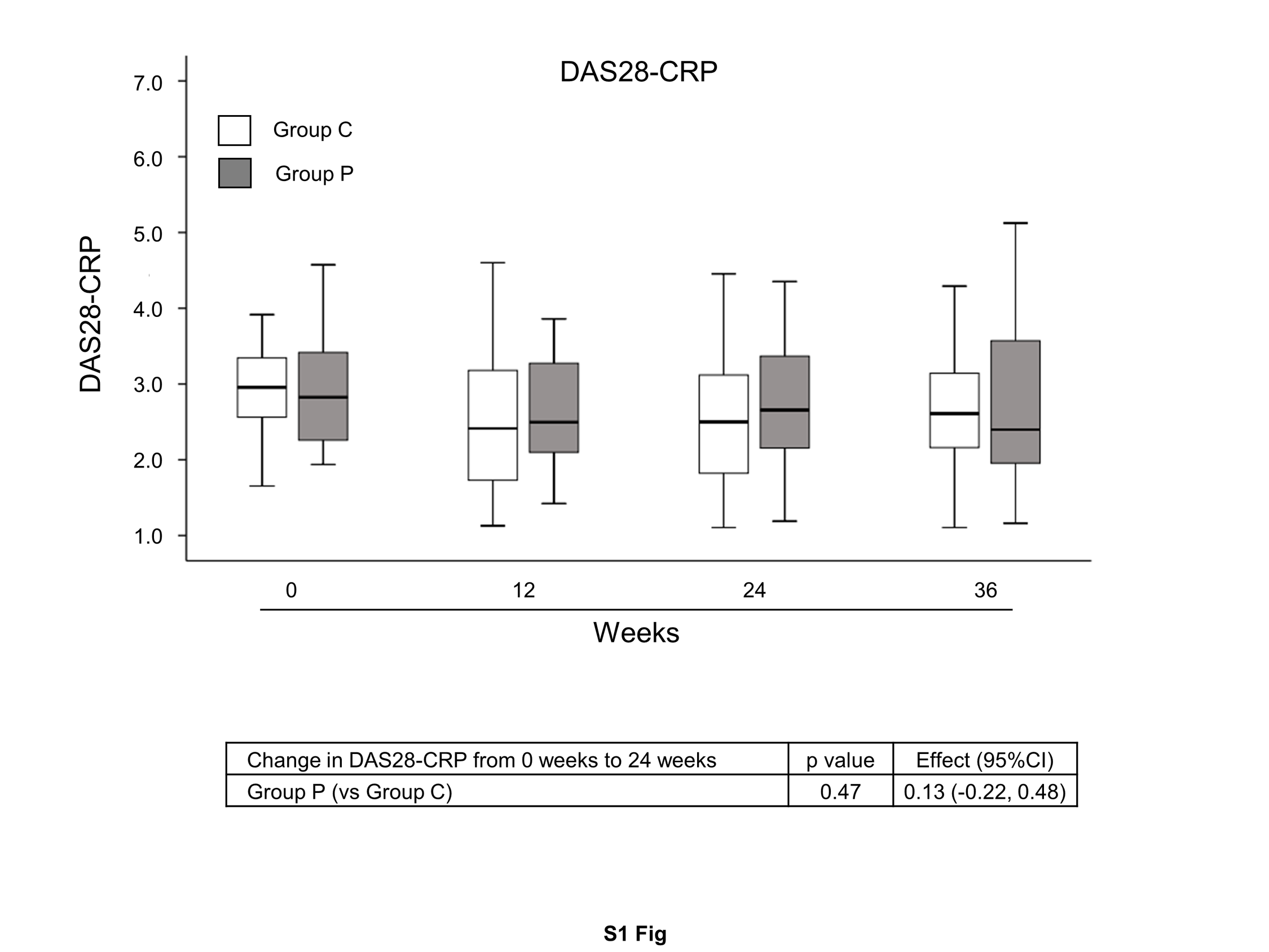

Supplement: S1 Fig — Data are presented as box-and-whisker plots. White box, Group C; gray box, Group P. P values are calculated using a mixed-effects model. C, control; CI, confidence interval; CRP, C-reactive protein; DAS, disease activity score; P, propolis. (TIF) [file pone.0252357.s001.tif]

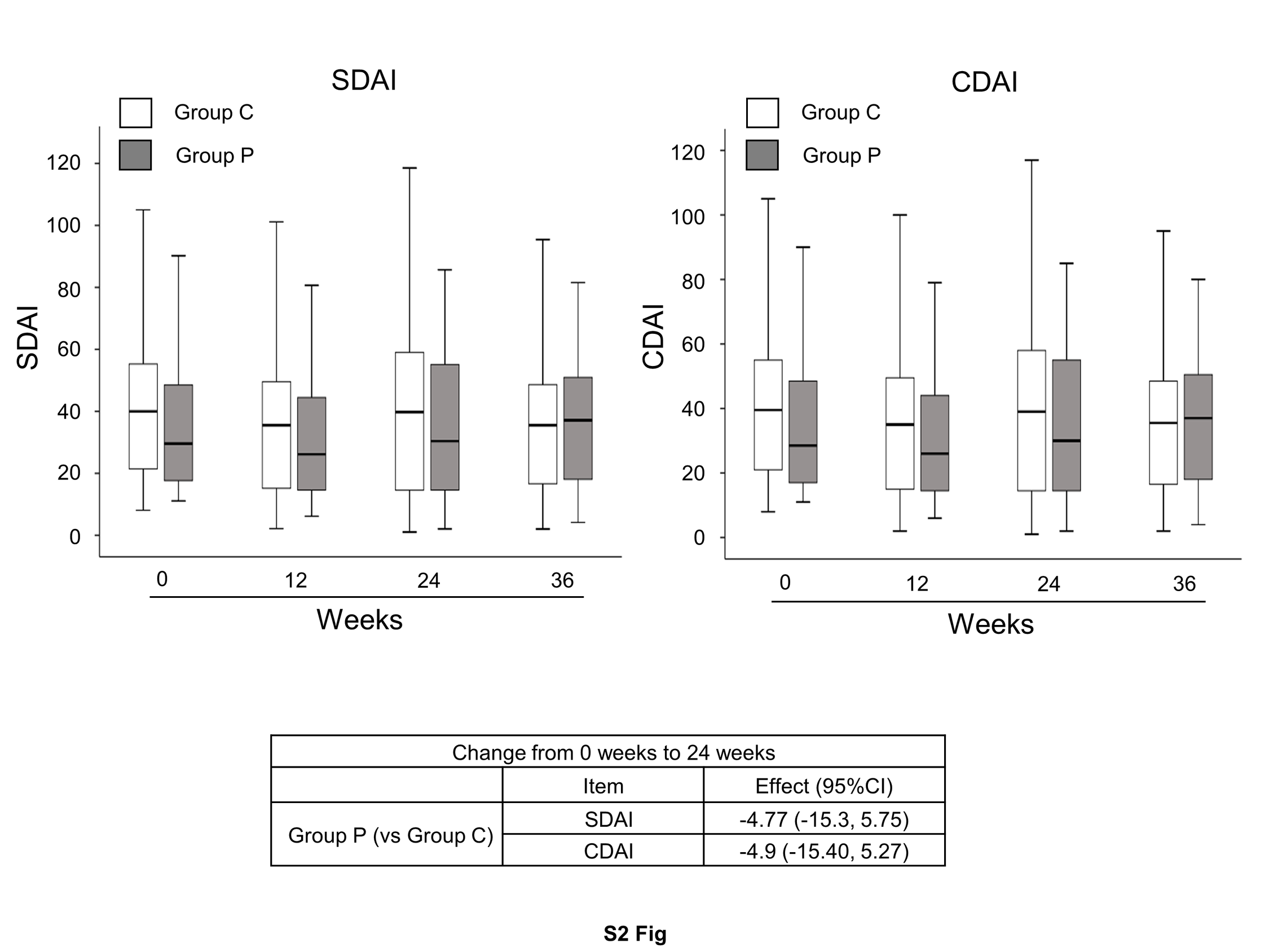

Supplement: S2 Fig — Data are presented as box-and-whisker plots. White box, Group C; gray box, Group P. Bootstrap confidence intervals are calculated because the normality of the residuals is problematic (repeated 1000 times). C, control; CDAI, clinical disease activity index; CI, confidence interval; SDAI, simplified disease activity index; P, propolis. (TIF) [file pone.0252357.s002.tif]

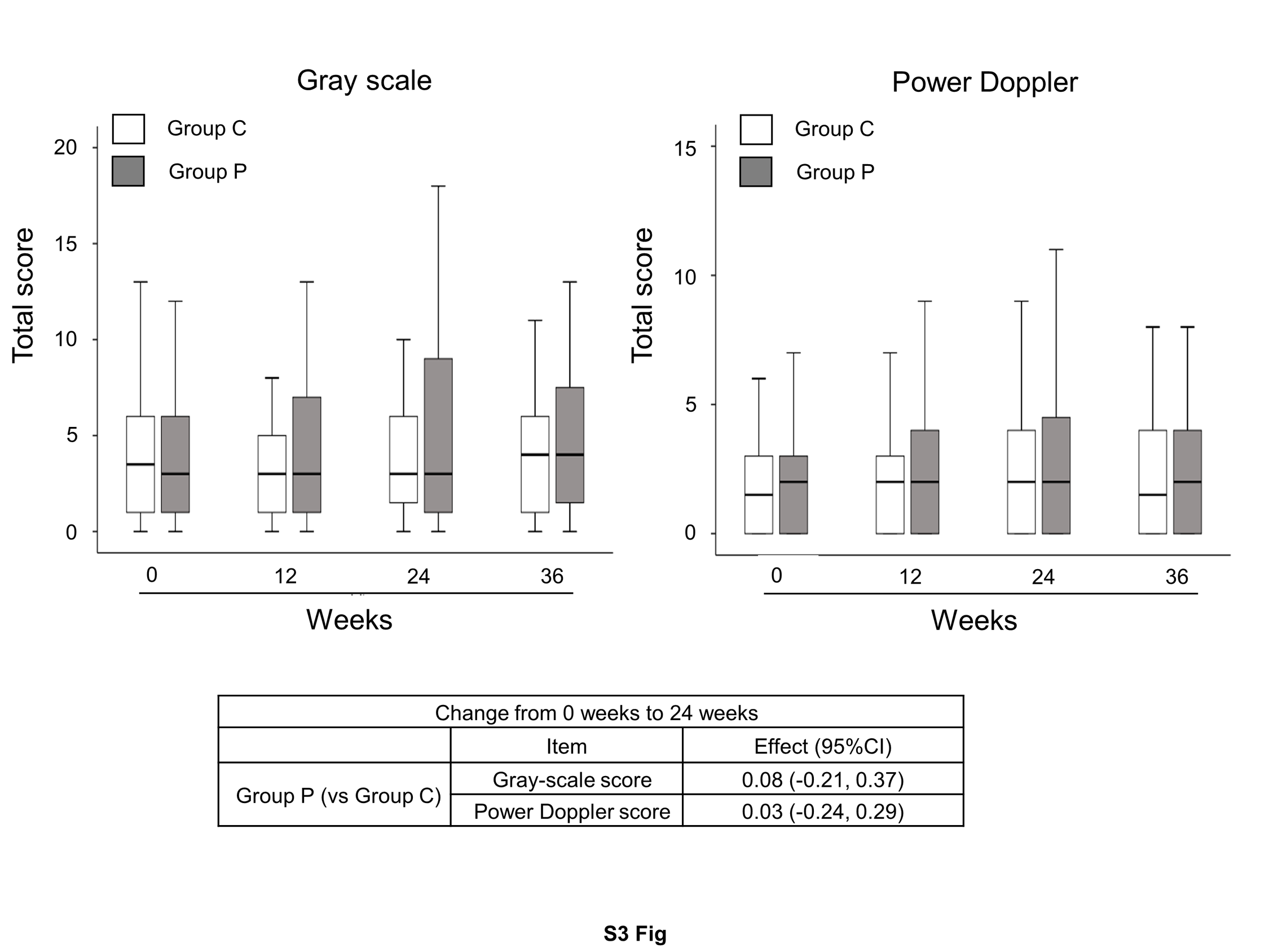

Supplement: S3 Fig — Data are presented as box-and-whisker plots. White box, Group C; gray box, Group P. Because the normality of residuals in the model was problematic, statistical analysis is performed after log-transforming total joint sonographic scores at each time point. P values are calculated using a mixed-effects model. C, control; CI, confidence interval; P, propolis. (TIF) [file pone.0252357.s003.tif]

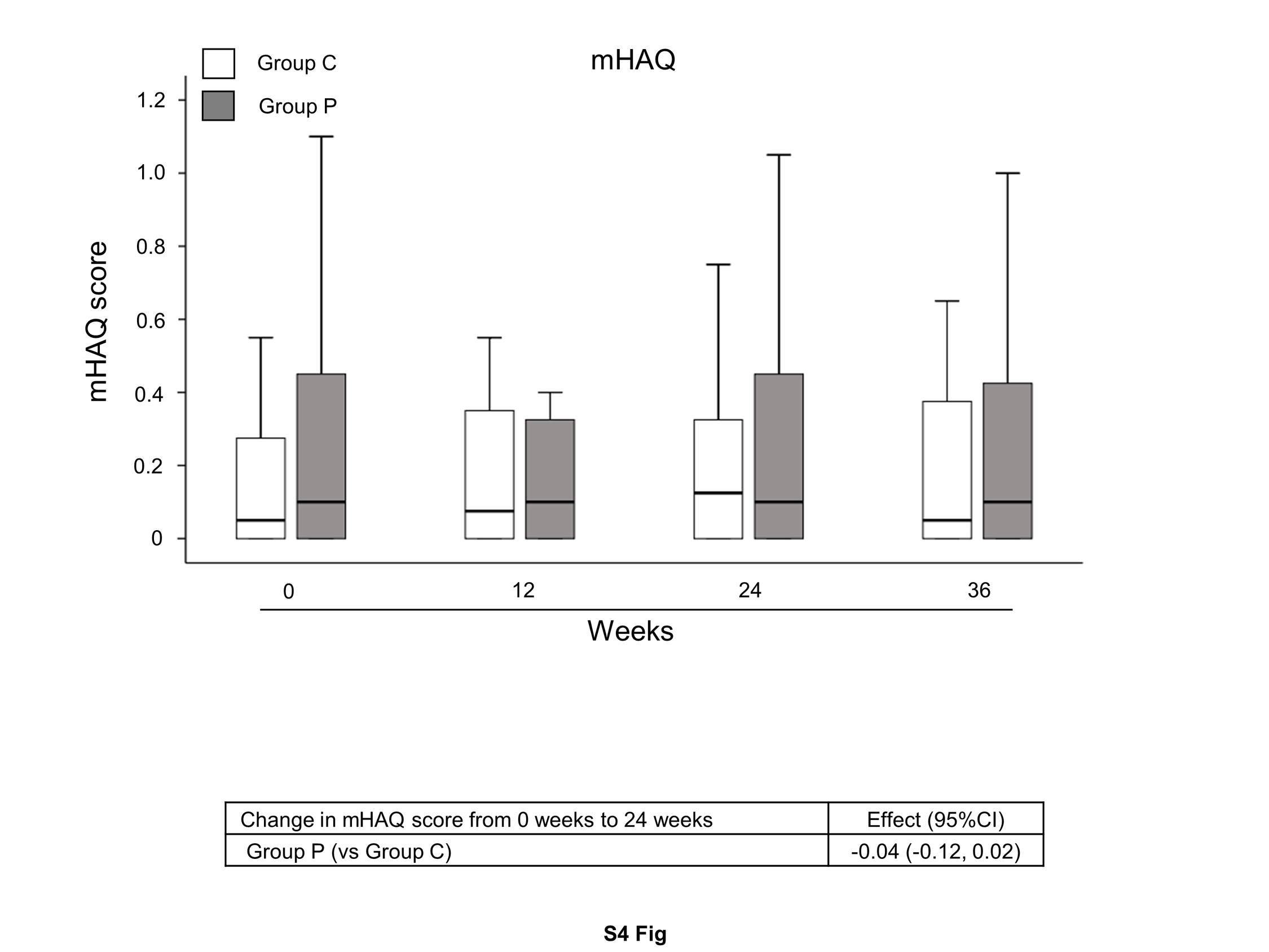

Supplement: S4 Fig — Data are presented as box-and-whisker plots. White box, Group C; gray box, Group P. Bootstrap confidence intervals are calculated because the normality of the model’s residuals is problematic (repeated 1000 times). C, control; CI, confidence interval; mHAQ, modified health assessment questionnaire disability index; P, propolis. (TIF) [file pone.0252357.s004.tif]
