# Supplementary material for: Double-blinded randomized controlled trial to reveal the effects of Brazilian propolis intake on rheumatoid arthritis disease activity index; BeeDAI
Source: PLoS One. 2021 May 27;16(5):e0252357. doi: 10.1371/journal.pone.0252357 (PMC8158978; doi:10.1371/journal.pone.0252357)
Supplement: S2 File — (DOCX) [file pone.0252357.s006.docx]

Double blinded randomized controlled trial to reveal the effects of Brazilian propolis intake to rheumatoid arthritis disease activity index (BeeDAI).

Study protocol plan

Research Representative

Tatsuya Koike Center for Senile Degenerative Disorders (CSDD),

Osaka City University Medical School, Abeno-ku, Asahimachi 1-4-3,

Osaka 545-8585, Japan

TEL: 06-6646-6010　FAX: 06-6646-6010

Principal Investigator

　Tatsuya Koike Osaka city university, Abeno-ku, Asahimachi 1-5-7,

Osaka 545-8586, Japan

TEL: 06-6646-6010　FAX: 06-6646-6010

First edition：31 Jan, 2018

# Outline

## Schema

randomized

REDCap

Female rheumatoid arthritis (RA) patients with moderate disease activity (DAS 28 - ESR is greater than 3.2, less than or equal to 5.1) n=80

Propolis group

n=40

Placebo group

n=40

36 weeks follow-up including post observation

- change in disease activity
- change in clinical test values
- change in sonographic examination of joints
- change in cytokines

primary endpoint

difference in DAS28-ESR change at 24 weeks

## Objective

　To investigate whether RA disease activity can be suppressed by feeding rheumatoid arthritis (RA) patients with propolis, a resinous mixture collected by honey bees from plant sources such as tree buds and sap.

## Subjects

　Eighty female RA patients who met the 2010 ACR/EULAR classification criteria, had moderate disease activity on DAS28-ESR assessment, and had no change in treatment for 3 months.

## Study method

　Eighty female RA patients with moderate disease activity according to the DAS28-ESR will be randomly divided into 40 patients in the propolis group (P group) and 40 patients in the placebo group (C group). P group takes 5 test tablets containing Brazilian propolis (508.5 mg as propolis) per day, and C group takes 5 placebo tablets without Brazilian propolis per day. The DAS28-ESR will be compared in both groups at the time of intervention and after 24 weeks. This will be followed up with a 12-week post-intervention observation period.

## Objective number of enrolled cases and study duration

Planned number of registered patients: 80

Registration period：After approval by the Ethics Review Committee 〜 31 March, 2019

Study period：After approval by the Ethics Review Committee 〜 31 December, 2019

Total study period：After approval by the Ethics Review Committee 〜 30 June, 2020

List of definitions for terms and abbreviations

Terms used in this study protocol

| Terms | Definitions |
| --- | --- |
| TOMORROW study | Total Management of Risk Factors in Rheumatoid Arthritis Patients to Lower Morbidity and Mortality study |

Abbreviations defined in this study protocol

| Abbreviations | Definitions |
| --- | --- |
| P group | Brazilian propolis containing test tablets administration group |
| C group | Brazilian propolis non-containing test placebo tablets administration group |

List of abbreviations for general items

| Abbreviations | Unabbreviated expressions |
| --- | --- |
| RA | rheumatoid arthritis |
| DAS | disease activity score |
| ESR | erythrocyte sedimentation rate |
| ACR | American College of Rheumatology |
| EULAR | European League against Rheumatism |
| IL | interleukin |
| QOL | quality of life |
| MTX | methotrexate |
| DMARDs | disease modifying anti-rheumatic drugs |
| bDMARDs | biological DMARDs |
| csDMARDs | conventional synthetic DMARDs |
| TNF-α | tumor necrosis factor-alpha |
| ADL | activities of daily living |
| CRP | c- reactive protein |
| SDAI | simplified disease activity index |
| CDAI | clinical disease activity index |
| mHAQ | modified health assessment questionnaire |
| WBC | white blood cells |
| RBC | red blood cells |
| Hb | hemoglobin |
| Plt | platelet |
| AST | aspartate transaminase |
| ALT | alanine aminotransferase |
| γ-GTP | γ-glutamyl transpeptidase |
| T-Bil | total bilirubin |
| Cre | creatinine |
| BUN | blood urea nitrogen |
| RF | rheumatoid factor |
| Alb | albumin |
| T-chol | total cholesterol |
| HDL-chol | high density lipoprotein cholesterol |
| TG | triglyceride |
| Na | sodium |
| Cl | chlorine |
| K | potassium |
| Mg | magnesium |
| P | phosphorus |
| Ca | calcium |
| FBS | Fasting blood sugar |
| CCP | cyclic citrullinated peptide |
| MMP | matrix metalloproteinase |
| SF-36 | 36-Item Short-Form Health Survey |
| SJC | swollen joints count |
| TJC | tender joints count |
| GH | global health assessment |
| VAS | visual analog scale |
| NSAID | non-Steroidal Anti- Inflammatory Drug |
| BMI | body mass index |
| REDCap | research electronic data capture |
| CRC | clinical research coordinator |

**Contents**

0 Outline 1

0.1 Schema 1

0.2 Objective 1

0.3 Subjects 1

0.4 Study method 1

0.5 Objective number of enrolled cases and study duration 2

　List of definitions for terms and abbreviations．．．．．．．．．．．．．．．．．．．．．．．．．．．．．．．．．．．．．．．．iii, iv

Contents．．．．．．．．．．．．．．．．．．．．．．．．．．．．．．．．．．．．．．．．．．．．．．．．．．．．．．．．．．．．．．．．．．．．．．．．．．v, vi, vii

1 Study background 1

2 Study aims 1

3 Overview of the test foods 2

3.1 Test foods 2

3.2 Anticipated adverse reactions and, in the case of medical devices, anticipated defects.…2

4 Diagnostic criteria and stage/type classification of target diseases 2

5 Subject patients 4

5.1 Inclusion criteria 4

5.2 Exclusion criteria 5

6 Case registration and assignment 5

7 Study plan 6

7.1 Study type and design 6

7.2 Study outline 6

7.3 Duration of subject's participation in the study 6

7.4 Dosage and administration of the test food, and duration of administration 6

7.5 Provisions on concomitant medications (therapies) 7

7.6 Methods of dose reduction and withdrawal (study medicine adjustment methods) 7

7.7 Control and delivery procedures for test foods 7

7.8 Intake guidance information 7

7.9 Actions after the study (or "post-treatment") 7

7.10 Preservation of samples, etc. and use of samples, etc. by other institutions, etc. 8

8 Observation and examination items and schedule 8

9 Discontinuation criteria for individual subjects 10

10 Handling of adverse events 10

10.1 Response to subjects in an adverse event 10

10.2 Serious adverse event reporting 11

10.3 Other adverse events 11

11 Termination, discontinuation, or suspension of the study 11

11.1 Termination of the study 11

11.2 Discontinuation, or suspension of the study 11

12 Evaluation items 11

12.1 Primary endpoints 11

12.2 Secondary endpoints 12

13 Data tabulation 12

14 Statistical analysis 12

14.1 Data handling 12

14.2 Efficacy analysis 12

14.3 Secondary analysis 13

14.4 Interim analysis 13

15 Objective number of cases and evidence of setting 13

15.1 Objective number of cases 13

15.2 Evidence of setting 13

16 Study period 14

17 Ethical issues 14

17.1 Compliance with the guidelines and Declaration of Helsinki 14

17.2 Approval of the Ethics Committee 14

17.3 Consent and explanation documents and provision of information to subjects 14

17.4 Consideration for human rights (protection of personal information) 15

17.5 Considerations for safety and disadvantage 15

17.6 Consultation and other support 15

18 Covering the cost of the study 16

18.1 Study funding and conflicts of interest 16

18.2 Patient's cost burden 16

18.3 Compensation and insurance coverage for health problems 16

19 Storage of records 16

20 Registration of study plans and publication of study results 16

21 Study organization 17

21.1 Research representative 17

21.2 Site and principal investigator 17

21.3 Study secretariat 17

21.4 Study coordination secretariat 17

21.5 Effectiveness and safety evaluation committee 17

21.6 Monitoring staff (monitor) 17

21.7 Data center 17

21.8 Biostatisticians 17

21.9 Clinical laboratories 17

22 Deviations or changes from the protocol 18

23 Monitoring/data quality control 18

24 Surveillance 18

25 References 18

26 Attachments/Appendixes 18

# Study background

　Rheumatoid Arthritis (RA) is an autoimmune disease whose true cause is unknown. It is thought

that an autoimmune response is initiated by exposure to environmental factors such as smoking and periodontal disease in addition to some genetic background. When an autoimmune response is

initiated, inflammation in the joints or systemically causes swelling and pain in the joints,

which progresses to destruction and deformity of the joints. In RA, osteoclasts are activated by

inflammatory cytokines, resulting in bone destruction. The prevalence of RA in adults is about 0.5-1% worldwide, and it occurs mostly in women in their 30s to 50s, with more than 700,000 patients in Japan. Despite this large number of RA patients, the detailed pathogenesis of RA remains

unknown, and there is currently no fundamental treatment strategy. Currently, the main treatments for RA symptoms such as joint pain, swelling, inflammation, and other disease activity include

pharmacotherapy and surgical treatments, with pharmacotherapy being the cornerstone of RA

treatment. Medications include disease-modifying anti-rheumatic drugs (DMARDs) such as

methotrexate (MTX), biologics that molecularly target inflammatory cytokines such as infliximab, a monoclonal antibody against TNF-α (tumor necrosis factor-alpha), and tocilizumab, an IL-6

(interleukin-6) receptor antibody. In addition, there are corticosteroids and other medicines that aim to reduce inflammation. The problems with medications are, of course, that they have various side effects, that biologics and other medicines are expensive and not available for all patients, and that they may not be equally effective for all patients. Therefore, many patients continue to receive treatment without reaching the current treatment goal of remission.

　The health food used in this study is a test meal containing Brazilian propolis from Yamada Bee Company, Inc. Propolis is a sticky resinous substance made by honeybees by collecting plant buds, sap, and pollen and mixing them with saliva and beeswax that they secrete. The main plant sources

of the propolis bulk of the extraction source of the Yamada Bee Company, Inc. Brazilian propolis-

containing test food is Baccharis dracunculifolia collected in the state of Minas Gerais, Brazil. For arthritis, Brazilian propolis has been reported to contribute to the suppression of disease

activity in a mouse rheumatoid arthritis model. As a mechanism, it has been reported that Brazilian propolis suppresses the phosphorylation of STAT3 (signal transducer and activator of

transcription 3), which is necessary for the differentiation process of naive helper T cells into IL-17 producing helper T cells (Th17 cells), and suppresses IL-17 production. Based on the above, propolis may contribute to the suppression of disease activity in RA, which may lead to the

development of new RA therapeutics. However, currently, there are only research results from

animal experiments in a mouse rheumatoid arthritis model, and the suppressive effect of propolis

on disease activity in humans is unknown. Therefore, in this study, we would like to clinically

examine how propolis administration changes the disease activity of RA patients. Since the disease activity index includes the patient's own evaluation of pain and disease activity, blinding is

essential, and we would like to prepare propolis-free test tablets and conduct them as a double-

blind randomized trial.

# Study aims

Object disease patients: 80 female RA patients who met the 2010 ACR/EULAR classification criteria. Test food: Test tablets containing Brazilian propolis

Evaluation items：

・Anthropometry: height, weight, waist circumference

・Disease activity：DAS28-ESR・DAS28-CRP・SDAI・CDAI

・ADL：mHAQ

・Laboratory data: General items（WBC, RBC, Hb, Ht, Plt, AST, ALT, γ-GTP, T-Bil, Cre, BUN, CRP, ESR, RF, TP, Alb, T-chol, HDL-chol, TG, Na, Cl, K, Mg, P, Ca, FBS, special items（anti CCP antibody, MMP-3, IL-6, IL-17, IL-10）

・Blood pressure

・Lifestyle survey: smoking status, alcohol consumption, exercise habits, sleep time, supplement use and type

・QOL:SF－36

・Objective arthritis assessment: sonographic examination of joints

　Eighty female RA patients with moderate disease activity by DAS28-ESR will be divided into two groups: 40 patients treated with Brazilian propolis (P group) and 40 patients treated with placebo balls (C group). In doing so, allocation will be by double-blind randomization using a stratified replacement block method, with facilities in strata. The test food is manufactured at Yamada Bee Company, Inc., Ltd. for 40 people in each group, plus 5 people in the reserve group, for a 3-month supply of 5 tablets per day x (84 days + 7 days in reserve) x 45 people = 2,0475 tablets. This is one set and we plan to make two sets for 6 months. The P group will take 5 tablets of the test diet containing Brazilian propolis (508.5 mg as P) per day, and the C group will take 5 tablets of the placebo diet without Brazilian propolis per day. In both groups, DAS28-ESR will be compared at 0, 12, 24, and 36 weeks, and the above evaluation items will also be compared.

The purpose of this study is to clarify whether Brazilian propolis has an suppressive effect on disease activity not only in mouse RA models but also in RA patients.

# Overview of the test foods

## Test foods

See the test food summary for details.

## Anticipated adverse reactions and, in the case of medical devices, anticipated defects

See the test food summary for details.

# Diagnostic criteria and stage/type classification of target diseases

The diagnosis of RA is based on the 2010 ACR/EULAR classification criteria.

The DAS28-ESR classification of disease activity for RA is as followsDAS28-ESR

Less than 2.6: Remission

2.6 or higher, 3.2 or lower: low disease activity

Greater than 3.2, less than 5.1: moderate disease activity

Greater than 5.1: High disease activity

DAS28-ESR = 0.56 × √28TJC + 0.28 × √28SJC + 0.76In(ESR) + 0.014 × GH-VAS

SJC and TJC are assessments of 28 joints in the body.

# Subject patients

　Female RA patients with moderately active disease (greater than or equal to 3.2 and less than or equal to 5.1) on the DAS28-ESR at the time of intervention. The inclusion criteria were patients with moderate disease activity, because the therapeutic goal for RA is at least low disease activity or less (DAS-ESR 3.2 or less), and higher disease activity (greater than 5.1) should be selected for more aggressive pharmacological treatment.

## Inclusion criteria

　Patients who meet all the following criteria should be selected for inclusion.

1. RA patients meeting the 2010 ACR / EULAR classification criteria (irrespective of disease duration)
2. Age: Patients who are between 30 and 70 years old at the time of obtaining consent
3. Gender：Female
4. Moderate disease activity (DAS28-ESR is greater than 3.2, less than or equal to 5.1)
5. After receiving sufficient explanation for the participation of this study, patients who obtained document consent by the patient's free will with sufficient understanding.
6. Administration of bDMARDs and csDMARDs is constant over 12 weeks before intervention
7. The dose of NSAID and steroid (predonisolone equivalent 10 mg / day or less) dose is also constant for 12 weeks or more before consent acquisition
8. Outpatient

［Evidence of setting］

1) Because this is a study of RA patients and the duration of illness is not expected to

affect the results of the study.

2) This is due to the epidemiological background of RA. Because most outpatients are between

30 and 70 years of age.

3) We restricted the study to females, much of the population, so as not to be influenced by

gender.

4) Because we believe that if the disease activity is below low disease activity, therapeutic target is reached, and high disease activity is not the target of the health food.

5）From an ethical perspective.

6)-7) To exclude the effect of medication on disease activity, as it has a strong impact on

patients treated with medications for RA.

8) To perform disease assessment every three months.

## Exclusion Criteria

Patients who meet any of the following criteria will be excluded.

1. Patients who cannot come to the examination on a regular basis
2. Patients with undifferentiated arthritis
3. Patients taking propolis as a supplement within 4 weeks before consent acquisition
4. Patients with significant obesity (BMI 30 or more)
5. Patients with abnormalities in liver function (AST or ALT more than 3 times higher than normal upper limit)
6. Patients with abnormal renal function (BUN 25 mg / dL or more or serum creatinine 2.0 mg / dL or more)
7. Patients who are pregnant or lactating
8. Patients with food allergy history
9. Others, patients judged by doctors as inappropriate as subjects

［Evidence of setting］

1) For impact on efficacy assessment and safety considerations

2) To evaluate the efficacy in RA patients

3)-4) Because of its impact on efficacy assessment

5)-9) For safety considerations

# Case registration and assignment

Assignment and coding of study foods: double-blind

After the test and placebo foods are manufactured at Yamada Bee Company, Inc., they are packaged at the Institute for Bee Products & Health Science in sachets containing five tablets for one day and 91 sachets for 84 plus seven days as a three-month supply for one person. The Institute for Bee Products & Health Science will then randomly assign study food allocation codes to the entire set (40 participants per group + 5 spares x 2 groups = 90 participants) and send the allocation list only to the REDCap randomization staff. In the Institute for Bee Products & Health Science, 1) All test tablets are coated with white color to ensure the indistinguishability of the test food. (2) The test food assignment code list will be kept by the Institute for Bee Products & Health Science, and REDCap test food assignment and coding staff, until the key is opened, and will not be disclosed to any other person. Keys will not be opened until the study is completed and the data for each case is fixed, except in the case of emergency evacuation. In addition, one unit (one set) of test food shall be used for one case.

Method of enrollment of subjects: Randomization

Prior to subject enrollment, the principal investigator or subcontracting physician will provide the person who is to be the subject of the study with an explanatory document that has been approved by each medical institution (the explanatory document in the Appendix or an explanatory document modified by each medical institution). Then, in principle, the contents of the "Ethical Guidelines for Medical Research Involving Human Subjects" will be explained in detail orally, and free and voluntary consent for participation in the study will be obtained in writing. When obtaining consent, the prospective subject should be given sufficient time and opportunity to ask questions to determine whether to participate in the study, and questions should be fully answered. After obtaining consent, the required fields are entered into the subject registration screen in the REDCap system to confirm eligibility. Once eligibility has been confirmed, random allocation of test subjects will be conducted by inputting information from the test food allocation screen in the REDCap system. The distribution of the test foods will be conducted on the same day.

Registration system: REDCap system

URL：ocu.jp/redcap

# Study plan

## Study type and design

The study design was a multicenter, double-blind, randomized, parallel-group, placebo-controlled trial. It is known as the method with the highest level of evidence in verifying the effects of foods.

## Study outline


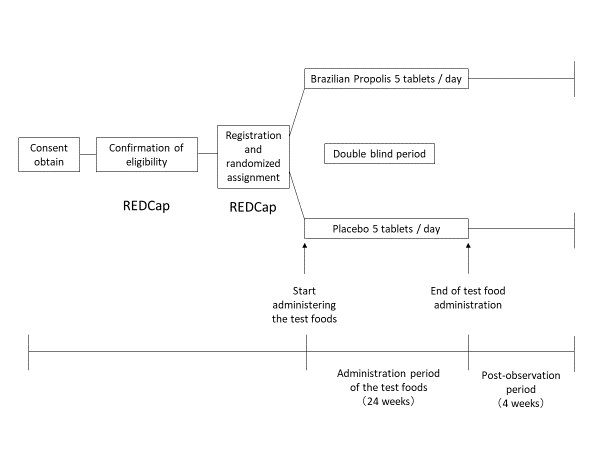


## Duration of subject's participation in the study

All patients up to 40 weeks follow-up.

## Dosage and administration of the test food, and duration of administration

　P Group will receive 5 test tablets containing Brazilian propolis daily, and C Group will receive 5 placebo tablets daily. No time of administration is specified. The test food will be provided in sachet packages of five tablets each, with the intention of ingesting five tablets at a time, but it will be acceptable for study participants to ingest them in divided portions. If patients do not take 5 tablets in a day, do not take them the next day and return the whole sachet at next visit. No more than five tablets per day were to be taken. The entire sachet will be collected at each visit, so any leftovers should be checked and recorded at this time. The administration period should be 24 weeks.

［Evidence for setting the dose and duration of administration］

　The dose of the test food containing Brazilian propolis used in this study was 5 tablets (508.5 mg of Brazilian propolis extract powder), which is the midpoint of the 4-6 tablets per day set as the standard dose by Yamada Bee Company, Inc.

## Provisions on concomitant medications (therapies)

１）Concomitant medications (therapy): No change in concomitant medications will be made if possible during the 24 weeks of the study. However, this does not apply if the disease

activity cannot be adequately controlled. Biologics can also be used in combination as

long as they are given at a constant dose starting 12 weeks before the intervention.

２）Prohibited medication (therapy): Propolis intake (purchased at own cost)

３）Restricted concomitant medications (therapy): None listed

４）Concomitant medications that should be used with caution (therapy): Warfarin potassium

５）Allowed concomitant medications (therapy): None listed

## Methods of dose reduction and withdrawal (study medicine adjustment methods)

　　　Since this study deals with food, there is no method for dose reduction or drug withdrawal.

## Control and delivery procedures for test foods

Each facility will receive a set of test foods and placebo in a ratio of 1:1, including spares, from the Institute for Bee Products & Health Science (26 for Osaka City University, 32 each for Shirahama Hamayu Hospital and Kitade Hospital, with the Kitade Hospital portion sent to Shirahama Hamayu Hospital). All of them are higher than the objective number of cases, so we believe there will be no shortage. Brazilian propolis tablets and placebo tablets will be managed by CRC. After randomization and allocation, a 12-week supply will be handed to each patient at the start of administration and at 12 weeks, respectively.

Storage location: Investigational product control room (for Osaka City University) and Search Institute for Bone and Arthritis Disease, Shirahama Foundation for Health and Welfare (for Shirahama Hamayu Hospital and Kitade Hospital)

Person responsible for storage: Tatsuya Koike, Principal Investigator

## Intake guidance information

- Time of intake: Not specified
- Intake method: Take 1-5 spheres with water without chewing.
- If forget to take a dose: skip that day's dose and the next day and thereafter, take as usual.
- Storage method: Store at room temperature
- How to return the remaining samples and containers: Bring them to the outpatient clinic at 12 weeks and 24 weeks.

## Actions after the study (or "post-treatment")

Strive to ensure that subjects receive the best possible prevention, diagnosis, and treatment based on the results of the study even after the study is completed.

## Preservation of samples, etc. and use of samples, etc. by other institutions, etc.

- Storage: Serum for cytokine assay will be stored at -80°C until assay at Osaka City University and Search Institute for Bone and Arthritis Disease.
- Method of use: All measurements are performed at LSI Medience, Inc. after collection ofall samples.
- Storage period: until measurement data is finalized
- Administrator: Tatsuya Koike, study supervisor
- Disposal method: Anonymized and disposed of after data is finalized
- Instruct and manage researchers, etc., to ensure that information, etc., is accurate,

and manage samples and information, etc., obtained from human subjects as necessary to prevent leakage, mixing, theft, loss.

- Report to the hospital director on the status of management in accordance with the procedure manual for the storage of samples and information obtained from human bodies.

# Observation and examination items and schedule

The following surveys will be conducted according to the timetable

1. Patient's background

　Medical record number, patient name, gender, date of birth, comorbidities, medical history, current medical history, previous treatment, concomitant medications (concomitant therapy and current treatment details), lifestyle (smoking status, alcohol consumption, exercise habits, sleep time, supplement use and type). However, medical record numbers and patient names are not entered into the REDCap (case report form).

1. Confirmation of intake of test foods

　The intake status during the study period will be assessed in percentage and recorded in REDCap. Participants will return all samples, including unused packaging materials, to the implementing medical institution at each visit. The CRC calculates the intake rate based on the number of tablets distributed and returned. For each case, the intake of the test food from the day after the previous visit to the day of the visit will be investigated and compliance with the intake will be requested.

1. Confirmation of subjective symptoms and other findings, evaluation of disease activity

Based on the questionnaire, palpation, and laboratory data, disease activity is quantified according to the following formula.

DAS28-ESR = 0.56 × √28TJC + 0.28 × √28SJC + 0.76In(ESR) + 0.014 × GH-VAS

DAS28-CRP = 0.56 × √28TJC + 0.28 × √28SJC + 0.36In(CRP × 10 + 1 ) + 0.014 × GH-VAS + 0.96

SDAI = 28TJC + 28SJC + PtGH-VAS(patient) + DrGH-VAS(physician) + CRP (mg/dl)

CDAI = 28TJC + 28SJC + PtGH-VAS(patient) + DrGH-VAS(physician)

1. Confirmation of adverse events and side effects: Describe in the medical record and case report the details, including abnormalities in various laboratory data, time of occurrence and disappearance, severity, treatment, outcome, assessment of severity, and relationship to the study food, and follow up as necessary. The severity is defined as 1) mild: administration can be continued without treatment, 2) moderate: administration can be continued with some treatment, and 3) severe: administration should be stopped or discontinued.
2. Height, weight, and waist circumference
3. Blood pressure and pulse rate
4. Hematological tests

WBC, RBC, Hb, Ht, Plt

1. Blood biochemistry test

AST, ALT, γ-GTP, T-Bil, Cre, BUN, CRP, ESR, RF, TP, Alb, T-chol, HDL-chol,TG, Na, Cl, K, Mg, P, Ca, FBS

1. Other specific tests

・anti-CCP antibody、MMP-3、IL-6、IL-17、IL-10

　Subcontractor name: LSI Medience Co.Ltd.

・QOL：SF－36

　　・ADL：mHAQ

・Disease activity：DAS28-ESR・DAS28-CRP・SDAI・CDAI

・Sonographic examination of joints：sonographic examination of synovitis in 7 joints on the side with stronger symptom at baseline (unchanged during the observation period) (2nd and 3rd metacarpophalangeal (MP) joints, 2nd and 3rd proximal interphalangeal (PIP) joints, wrist, and 2nd and 5th metatarsophalangeal (MTP) joints) using both grayscale (GS) (grade 0–3) and power-Doppler (PD) scale (grade 0–3)

Timetable

| Item | pre-  observation | Start administering the test foods | Administration period | End of test food administration | post-  observation |
| --- | --- | --- | --- | --- | --- |
| Period | Before  1-4 weeks | 0 week | After 12 weeks  (±7day) | After 24 weeks  (±7day) | After 12 weeks |
| Medical Examination | 1 | 2 | 3 | 4 | 5 |
| Consent obtain | ○ |  |  |  |  |
| Check patient background | ○ |  |  |  |  |
| Test food administration |  |  |  |  |  |
| Subjective symptoms and other findings | ○ | ○ | ● | ● | ● |
| Observation of adverse events^a^ |  |  | ● | ● | ● |
| Anthropometry^b^ |  | ○ |  | ● |  |
| Blood pressure and pulse measurement |  | ○ | ● | ● | ● |
| Hematological examination |  | ○ | ● | ● | ● |
| Blood Biochemistry Test |  | ○ | ● | ● | ● |
| Sonographic examination of joints |  | ○ | ● | ● | ● |
| QOL assessment |  | ○ |  | ● |  |
| Disease activity assessment^c^ | ○ | ○ | ● | ● | ● |

　Items marked with an asterisk ○ are items to be performed before administration of the test food, and items marked with an ● are items to be performed after administration of the test food.

a: Adverse events are all events that are undesirable, such as side effects, regardless of whether they are causally related to the test food.

b: Measure height, weight, and abdominal circumference as anthropometry.

c: Evaluate DAS28-ESR, DAS28-CRP, SDAI, and CDAI for disease activity.

Blood tests should be performed under fasting conditions to rule out the effects of diet.

# Discontinuation criteria for individual subjects

　　The principal investigator or sub-investigator will discontinue the study for subjects who are unable to continue the study due to the following reasons, and will take appropriate treatment based on the benefit to the subject. In addition, scheduled surveys/inspections shall be conducted as soon as possible to make a decision (Except in cases where it cannot be conducted due to withdrawal of consent, etc.). If consent is withdrawn after the administration of the study food has begun, it should be made as clear as possible whether the withdrawal is due to ineffectiveness of the study food or an adverse event, or whether it is due to an accidental event (e.g., change of location), and it should be recorded so that it can be used as a reference for the selection of the case for efficacy and safety evaluation. The investigator or sub-investigator should record the date of discontinuation, reason for discontinuation, and comments in the case report form. After discontinuation of the study, if any of the following items 4)-6), 10) are found, hematological and biochemical tests should be performed to confirm safety. In addition, medical examinations will be conducted as deemed necessary by the physician for each symptom. Patients will be followed up for one year after treatment as deemed necessary by the physician according to the examination results. In particular, if the study is discontinued due to the occurrence of an adverse event, follow-up will be conducted until the patient recovers to the original state as much as possible.

1. When the subject declines to participate in the study or withdraws consent
2. Other reasons depending on the subject (e.g., too busy, hospital transfer, relocation)
3. If it is found that the eligibility is not satisfied after registration
4. When it is judged inadvisable to continue administration of the test food due to worsening of the primary disease.
5. If it is difficult to continue the study due to exacerbation of complications
6. If it is difficult to continue the study due to adverse events
7. When pregnancy is detected
8. If the entire study is discontinued
9. If the physician deems it appropriate to stop the study for any other reason.

# Handling of adverse events

## Response to subjects in an adverse event

　An "adverse event" is any unfavorable or unintended sign, symptom, or illness that occurs when a test food is administered, regardless of whether it is causally related to the test food.

When an adverse event is recognized, the principal investigator or sub-investigator should immediately take appropriate treatment and record it in the medical record and case report without any discrepancy.　In addition, subjects will be informed if the administration of the test food is discontinued or if treatment for adverse events becomes necessary. If it is necessary to identify the test food as an emergency evacuation, request the person in charge of test food allocation and coding through the principal investigator, etc., to disclose the results of opening the package for the case. When the occurrence of a serious adverse event is recognized, all serious adverse events will be promptly reported to the hospital director, regardless of whether there is a causal relationship with the research

## Serious adverse event reporting

　A "serious adverse event" is defined as any unfavorable medical event, regardless of dose, that meets the following criteria

(1) Lethal events

(2) Life threatening

(3) Events that require hospitalization for treatment

(4) Permanent or significant disability or malfunction

(5) Those that cause congenital anomalies in the offspring

If the principal investigator recognizes the occurrence of a serious adverse event while conducting an invasive study, the principal investigator shall promptly report all serious adverse events to the hospital director (Clinical Trial Review Committee in the case of Osaka City University), regardless of whether or not there is a causal relationship with the study, and shall also report to the responsible physician of the other medical institution if a causal relationship between the study and the serious adverse event cannot be ruled out in a multicenter study.

## Other adverse events

Other adverse events should be described in the case report form according to the procedures described in "8. Observation and examination items (4) Confirmation of adverse events and side effects.

# Termination, discontinuation, or suspension of the study

## Termination of the study

At the end of the study at each institution, the principal investigator shall promptly submit a study termination report to the hospital director. If the study is to be conducted at multiple institutions, a termination report will also be submitted to the principal investigator.

## Discontinuation, or suspension of the study

　The principal investigator will consider whether to continue the study if any of the following apply.

1) When significant information regarding the quality, safety, or efficacy of the test food is obtained.

2) When it is judged to be difficult to recruit subjects and to achieve the planned number of cases.

3) When the Review Committee instructs changes to the implementation plan, etc., and it is judged to be difficult to accept the changes.

If the Review Committee recommends or directs that the study be stopped, the study will be stopped. In the case of a multi-center study, the principal investigator or the committee specified in the study protocol will review the above items and consider whether to continue the study. When a decision is made to discontinue or suspend a study, the decision shall be promptly reported to the hospital director in writing with the reasons for the decision.

# Evaluation items

## Primary endpoints

　The primary endpoint of this study is the change in disease activity DAS28-ESR at 24 weeks after the start of the test food intake.

【Evidence of setting】

　In this study, we will investigate the suppressive effect of Brazilian propolis on the disease activity of RA. The above item of disease activity is the gold standard not only for ACR/EULAR but also for the assessment of disease activity in RA worldwide.

## Secondary endpoints

1. DAS28-CRP, SDAI and CDAI at week 12, 24 and 36.
2. QOL evaluated by the change of the score by SF-36 at week 24.
3. The change of ADL evaluated by mHAQ at week 12, 24 and 36.
4. The change of sonographic total scale of hand and foot joint at week 12, 34 and 36.
5. Adverse events requiring discontinuation of the test food up to 24 weeks after starting ingestion.
6. Severe adverse events up to 36 weeks after starting ingestion.
7. All adverse events up to 36 weeks after starting ingestion.

【Evidence of setting】

1） Other methods of assessing disease activity other than the DAS28-ESR, each of which has its own advantages and disadvantages, should also be used.

2) As a general QOL assessment tool for subjects, the SF-36 has been reported in numerous articles due to its simplicity and reliability. We will evaluate the effect of QOL improvement associated with suppression of disease activity.

3) It is the gold standard for the assessment of ADL in RA patients not only in ACR/EULAR but also worldwide. We will evaluate the improvement of ADL with the suppression of disease activity.

4) Obtain a more objective assessment because disease activity assessment includes many subjective items.

5-7) To evaluate the safety of test foods.

# Data tabulation

All data is collected by entering the required fields into the eCRF (electronic case report form) built into the REDCap system. In principle, each visit should be entered within 7 days of the subject's visit to the hospital. Since there are no entry fields for personal information such as medical record numbers, a separate correspondence chart that identifies individuals should be prepared and kept strictly by the principal and subcontracting physicians.

# Statistics Analysis

## Data handling

When compiling data for observation and inspection items specified in the plan by time period, data for inspection items performed on days or at times outside the acceptable range shall be treated as missing. For missing values, LOCF completes the values when the main analysis is performed.

## Efficacy analysis

The analysis population will be defined as the largest analysis set (full analysis set; FAS), defined as subjects assigned to the study who have consumed the study food at least once and have had at least one efficacy assessment after administration of the study food. Supplemental analyses will also be performed with a per protocol set (PPS) that conforms to the protocol. Hypothesis testing will be two-tailed at the 5% significance level, with two-tailed 95% confidence intervals.

A significance test for the coefficients of the groups will be conducted using a model in which the DAS28-ESR at the end of the intervention for the primary endpoint is the objective variable, the group (control or intervention) is the explanatory variable, and the value of the DAS28-ESR at week 0 is the covariate. In order to confirm the robustness of the results associated with the completion of missing values by LOCF, a mixed-effects model will be used as a supplementary analysis for the primary endpoint. In this case, the normality of the DAS28-ESR at the end of the intervention is checked by histogram, and if normality is not found, the variable is transformed by log transformation or other methods to follow a normal distribution before being used in the analysis. As a secondary analysis, when the objective variable is DAS28-ESR, the interaction between group (control, intervention) and time point (12 weeks, 24 weeks) and between group and time point will be used as explanatory variables, and the analysis will be conducted using a mixed-effects model with the value of DAS28-ESR at week 0 as the covariate and subjects as the variable effect, and the correlation structure of time points will be unstructured.

## Secondary analysis

Analyses of secondary endpoints will be conducted to provide supplementary insights to the primary analyses of the study. The secondary endpoints will be analyzed with exploratory intent and will not be adjusted for multiplicity. 1) DAS28-CRP, SDAI, CDAI, at 12, 24, and 36 weeks after intake 2) QOL at 24 weeks after intake (SF-36), 3) ADL at 12, 24, and 36 weeks after intake: mHAQ 4) sonographic examination of joints (scale change) at 12, 24, and 36 weeks after the start of intake will be analyzed in the same way as the primary endpoint. A frequency table will be prepared for each administration group regarding the occurrence of adverse events. In the case of serious adverse events, Kaplan-Meier curves will be generated for survival analysis until the occurrence of the event, and the groups will be compared using Cox proportional hazards model with DAS28-ESR at 0 week as a covariate. For quantitative data on clinical laboratories, summary statistics will be calculated for each time point and the amount of change from before the start of the test food administration for each dose group, and a scatter plot will be created. For other qualitative data, a frequency table should be created for each administration group.

## Interim analysis

The food to be administered is a health food that is already sold worldwide and is unlikely to cause serious adverse events. In addition, it is not a powerful drug, so it is unlikely that the effect will appear in a short period of time. Since the total administration study period is 24 weeks, we believe that there is little merit in conducting an interim analysis and will not conduct an interim analysis.

# Objective number of cases and evidence of setting

## Objective number of cases

Number of participating institutions: 3 institutions, Objective number of cases: 80

## Evidence of setting

　There are no clinical trials in which propolis containing this test food has been administered to patients with RA. In a similar study examining the effect of Mediterranean diet intervention on RA patients (Ann Rheum Dis 2003: 62: 208-214), the mean difference in DAS28-ESR between 25 patients in the control group and 26 patients in the intervention group at 12 weeks was 0.4 points (statistically significant difference between the two groups at 12 weeks). However, the study did not use the DAS28-ESR as a subject eligibility criterion, and there was concern about the large variability in disease activity among study participants. In this study, the entry criterion is limited to moderately active disease from the viewpoint of clinical disease management, and the intervention effect of the test food on DAS28-ESR is expected to be the same as or greater than that of the above study. Therefore, the following hypotheses were designed based on the data from the cohort study of RA patients and non-RA controls that has been conducted in Japan since 2010 by a research group including the principal investigator. Of the 182 rheumatoid arthritis patients who continued to participate in our cohort study, the TOMORROW Study, as of 2016, 67 RA patients with moderately active disease had a mean change in disease activity of -0.22 under standard treatment over 1 year. We assume that the add-on effect of propolis is -0.5. Assuming σ (standard deviation of expected outcome) = 0.78, at least 39 subjects in each group would be required to detect δ (the value we would like to see as the difference in DAS28-ESR after 24 weeks between the two groups) = 0.5 at a significance level of α = 0.05 with 80% power by a two-sample t-test. In consideration of the burden on subjects to participate in the study, we set the objective to have 40 subjects in each group for a total of 80 subjects, considering that there would be few dropouts during the study period.

# Study period

Registration period：After approval by the Ethics Review Committee 〜 31 March, 2019

(Registration deadline: 31 March, 2019)

Follow-up period：After approval by the Ethics Review Committee 〜 31 December, 2019

# Ethical issues

## Compliance with the guidelines and Declaration of Helsinki

All researchers and research collaborators involved in this study shall comply with the spirit of the Declaration of Helsinki (translated by the Japanese Medical Association, Fortaleza, Brazil, 2013). In addition, this study will be conducted in accordance with this study protocol and the "Ethical Guidelines for Medical Research Involving Human Subjects" (Ministry of Education, Culture, Sports, Science and Technology and Ministry of Health, Labour and Welfare Notification No. 3, 2014, partially revised on February 28, 2017).

## Approval of the Ethics Committee

This study will be conducted with the approval of the Review committee of Osaka City University hospital, the Ethics committee of Shirahama Hamayu hospital and the Ethics committee of Kitade Hospital.

## Consent and explanation documents and provision of information to subjects

Explanatory documents and consent documents that have been approved by the review committee of each institution are given to the patient, sufficient explanations are given in writing and orally, and the patient's free and voluntary consent is obtained in writing. When information on efficacy or safety is obtained that may affect the patient's consent, or when changes are made to the implementation plan that may affect the patient's consent, information is promptly provided to the patient and the patient's will is confirmed in advance as to whether participate in the study. In addition, revise the explanatory and consent documents, etc., with the prior approval of the review committee, etc., of each facility, and obtain the patient's re-consent.

The following items shall be included in the explanatory document

1. Introduction: About independent clinical trials

2. Your medical condition

3. Objectives of this clinical trial

4. Method of this clinical trial

5. Expected duration of participation in this clinical trial

6. Anticipated benefits and risks

7. Treatment options and what to do if you do not participate in this trial

8. Cost of treatment

9. We reserve the right to discontinue this trial.

10. What you must do if you agree to participate in this trial

11. In case of any health concerns during this trial

12. Intellectual property rights and conflicts of interest arising from research

13. Your participation in this study is of your own free will (including, in the case of equipment testing, matters related to the handling of the equipment in the event you decide not to participate in the study).

14. We will keep you informed about this trial.

15. Privacy must be maintained if the results of this trial are made public.

16. If you participate in this study, your medical records and other information may be reviewed during or after the trial.

17. Method of preservation, use after completion of research, or disposal of materials (including samples)

18. Consultation desk

## Consideration for human rights (protection of personal information)

The human rights, welfare, and safety of subjects shall be ensured to the maximum extent possible when conducting research. The principal investigator or sub-investigator should carefully consider ethical considerations when conducting this study, and obtain the consent of the patient in writing after fully explaining the contents of the study. Whether or not to participate in the study is determined by the free will of the subject himself/herself, and can be discontinued at any time by the subject himself/herself, even after consent. In addition, no disadvantage will be suffered due to the cancellation of participation. The information obtained in this study will not be disclosed in any form in which individuals can be identified under any conditions, and will be kept under strict control. In addition, if the individual wishes, information about him/her shall be reported in writing only to him/her.

## Considerations for safety and disadvantage

In the case of an adverse event, appropriate medical examination and treatment should be performed promptly. If there is no improvement and moderate to severe adverse events persist, discontinue the study for the subject.

## Consultation and other support

Respond appropriately and promptly to consultations, inquiries, complaints, etc., from research subjects, their substitutes, etc., and related persons.

# Covering the cost of the study

## Study funding and conflicts of interest

＜Conflicts of interest in research funding and research organizations＞

This study was funded in part by a grant of 2,000,000 yen from the Yamada Research Grant, which was applied for and received by Yoshinari Matsumoto, a co-investigator of this study (this research grant will be managed by the Shirahama Medical Welfare Foundation, a collaborative study institution). However, since this is a clinical trial on human subjects, Tatsuya Koike, a licensed physician (co-researcher since the time of application for the research grant), has been appointed as the principal investigator and has received research funding. For the test food, propolis test tablets and placebo will be provided free of charge from Yamada Bee Company, Inc. Yamada Bee Company, Inc. will provide information on the test foods, but will not be involved in the analysis of the study. The shortage of research funds will be covered by the self-research funds (e.g., scholarship donations) of the Department of Center for Senile Degenerative Disorders.

＜Conflicts of Interest of Researchers.＞

Although the Yamada Research Grant has provided research support for this study (provision of test tablets), the principal investigator and sub-investigators of this study have no other conflicts of interest to disclose regarding this study. The Principal Investigator will continuously check with the investigators (e.g., once a year at the beginning of each fiscal year) to ensure that no new "conflicts of interest" have arisen in the planning, implementation, and reporting of this study that may affect the results and interpretation of the results, and that the conduct of the study does not compromise the rights and interests of subjects.

## Patient's cost burden

This study will be conducted entirely within the scope of insurance coverage. Tests that are not covered by insurance will be performed at the expense of the study. In addition, the number of visits to the hospital and the frequency of examinations conducted by the subjects are comparable to those of normal medical treatment, so there is no increase in the burden on the subjects due to participation in the study. In addition, there is no reward for participating in the study.

## Compensation and insurance coverage for health problems

# Except for special tests (SF-36 and cytokine tests), the tests planned to be performed in this study are those that are performed at the same frequency in daily practice regardless of whether the patient participates in the study, and all tests will be performed within the range of insurance coverage. In the event of health problems arising from this study, treatment will be provided using the patient's health insurance as in the case of normal insurance treatment. Neither the organization/facility involved in this study nor any individual such as a physician will compensate the subjects for the payment of out-of-pocket medical expenses, compensation for absence from work, or compensation for the cost of a differential hospital stay in such cases. However, if the causal relationship is clear and health issues (death or severe disability) above a certain level occur, compensation will be provided by clinical research compensation insurance. As for the clinical research indemnity insurance, we are planning to conclude a contract with a coverage amount of 100 million yen per person and 300 million yen per accident.

# Storage of records

Since this study is classified as an interventional study involving invasion, it will be appropriately retained for at least five years after the date of the report on the completion of the study or three years after the date of the final publication of the results of the study, whichever is later.

# Registration of study plans and publication of study results

　Prior to initiating subject enrollment, the contents of the study plan are registered in the public registration system UMIN. The results will be published in appropriate academic meetings and medical journals. When the final publication of the results is made, it will be reported to the hospital director without delay.

# Study organization

## Research representative

Tatsuya Koike: Center for Senile Degenerative Disorders, Osaka City University Medical School

　Contract Professor　+81-6-6646-6010

## Institution and principal investigator

Center for Senile Degenerative Disorders, Osaka City University hospital

Contract Professor, Tatsuya Koike　+81-6-6646-6010

Shirahama Hamayu hospital, Orthopaedic Surgery, Tatsuya Koike, chief director +81-739-43-6200

Kitade hospital　Department of Rheumatology, Tatsuya Koike, part-time physician +81-738-22-2188

## Study secretariat

Center for Senile Degenerative Disorders, Osaka City University Medical School

Tatsuya Koike（Study secretariat representative）,Tomoko Nakatsuka

〒545-8585　 Abeno-ku, Asahimachi 1-4-3, Osaka 545-8585, Japan

+81-6-6646-6010

## Study coordination secretariat

　　No specific installation

## Effectiveness and safety evaluation committee

　　No specific installation

## Monitoring staff (monitor)

　　Center for Senile Degenerative Disorders, Osaka City University Medical School, Tomoko Nakatsuka

## Data center

　REDCap input will be used to create the case report form.

REDCap Management: Center for Senile Degenerative Disorders, Osaka City University Medical School, Adjunct Assistant Professor Yuko Sugioka

## Biostatisticians

　　 Department of Medical Statistics, Osaka City University Medical School, Professor Ayumi Shintani

## Clinical laboratories

　　LSI Medience Corporation.

　　Commissioned service: Measurement of IL-10, IL-17, IL-6, MMP-3, and anti-CCP antibodies

Supervision method: No storage at the company, as the sample is given after the final blood collection of the final entries is completed. The data is checked for reliability at the final stage.

# Deviations or changes from the protocol

When revising the implementation plan or explanatory and consent documents, prior approval from the Ethics Committee is required. However, this does not apply to changes that are medically unavoidable, such as those to avoid immediate danger to the subject.

If there is a significant deviation from the study protocol, the principal investigator or sub-investigator shall record the deviation along with the reason for the deviation.

The principal investigator shall revise the protocol as necessary when he/she becomes aware of matters related to the quality, efficacy, and safety of the test food or other information that is important for the correction of the study. In case of serious deviations from the efficacy study protocol, the deviations shall be recorded along with the reasons for them.

# Monitoring/data quality control

In this study, institutional visit monitoring may be conducted for the purpose of education and improvement of the scientific and ethical quality of the study. By confirming the items required by the principal investigator, the monitor assures that the clinical research is being conducted appropriately, that the necessary items are being recorded accurately, and that the reliability of the data is being maintained adequately. Center for Senile Degenerative Disorders, Osaka City University Medical School, which is the study secretariat, will be in charge of monitoring when conducted in this study.

# Surveillance

No plans to implement.

# References

Aletaha D, Neogi T, Sikman AJ, Funovits J, Felson DT, Bingham CO, et al. 2010 Rheumatoid arthritis classifi cation criteria: an American College of Rheumatology/European League Against Rheumatism collaborative initiative. Ann Rheum Dis 69:1580–1588, 2010

[Tanaka M](https://www.ncbi.nlm.nih.gov/pubmed/?term=Tanaka%20M%5BAuthor%5D&cauthor=true&cauthor_uid=21861090), [Okamoto Y](https://www.ncbi.nlm.nih.gov/pubmed/?term=Okamoto%20Y%5BAuthor%5D&cauthor=true&cauthor_uid=21861090), [Fukui T](https://www.ncbi.nlm.nih.gov/pubmed/?term=Fukui%20T%5BAuthor%5D&cauthor=true&cauthor_uid=21861090), [Masuzawa T](https://www.ncbi.nlm.nih.gov/pubmed/?term=Masuzawa%20T%5BAuthor%5D&cauthor=true&cauthor_uid=21861090).: Suppression of interleukin 17 production by Brazilian propolis in mice with collagen-induced arthritis. Inflammophamacology 20:19-26, 2012

Skoldstam L., Hagfors L., Johansson G.: An experimental study of a Mediterranean diet intervention for patients with rheumatoid arthritis. Ann Rheum Dis 62:208-214, 2003

# Attachments/Appendixes

None in particular.
