# Supplementary material for: Double-blinded randomized controlled trial to reveal the effects of Brazilian propolis intake on rheumatoid arthritis disease activity index; BeeDAI
Source: PLoS One. 2021 May 27;16(5):e0252357. doi: 10.1371/journal.pone.0252357 (PMC8158978; doi:10.1371/journal.pone.0252357)
Supplement: S3 File — (PDF) [file pone.0252357.s007.pdf]

二重盲検無作為化比較対照による
ブラジル産プロポリス摂取の関節リウマチ疾患活動性への
影響確認試験

Double blinded randomized controlled trial to reveal the effects of Brazilian propolis intake to rheumatoid arthritis disease activity index (BeeDAI).

実施計画書

研究代表者

小池　達也　　大阪市立大学大学院医学研究科高齢者運動器変性疾患制御講座
〒545-8585　大阪市阿倍野区旭町 1-4-3
TEL：06-6646-6010　FAX：06-6646-6010

試験責任医師

小池　達也　　大阪市立大学医学部附属病院
〒545-8586 大阪市阿倍野区旭町 1-5-7
TEL：06-6646-6010　FAX：06-6646-6010

初版：2018 年　1 月　31 日

0　概要

0.1　シェーマ

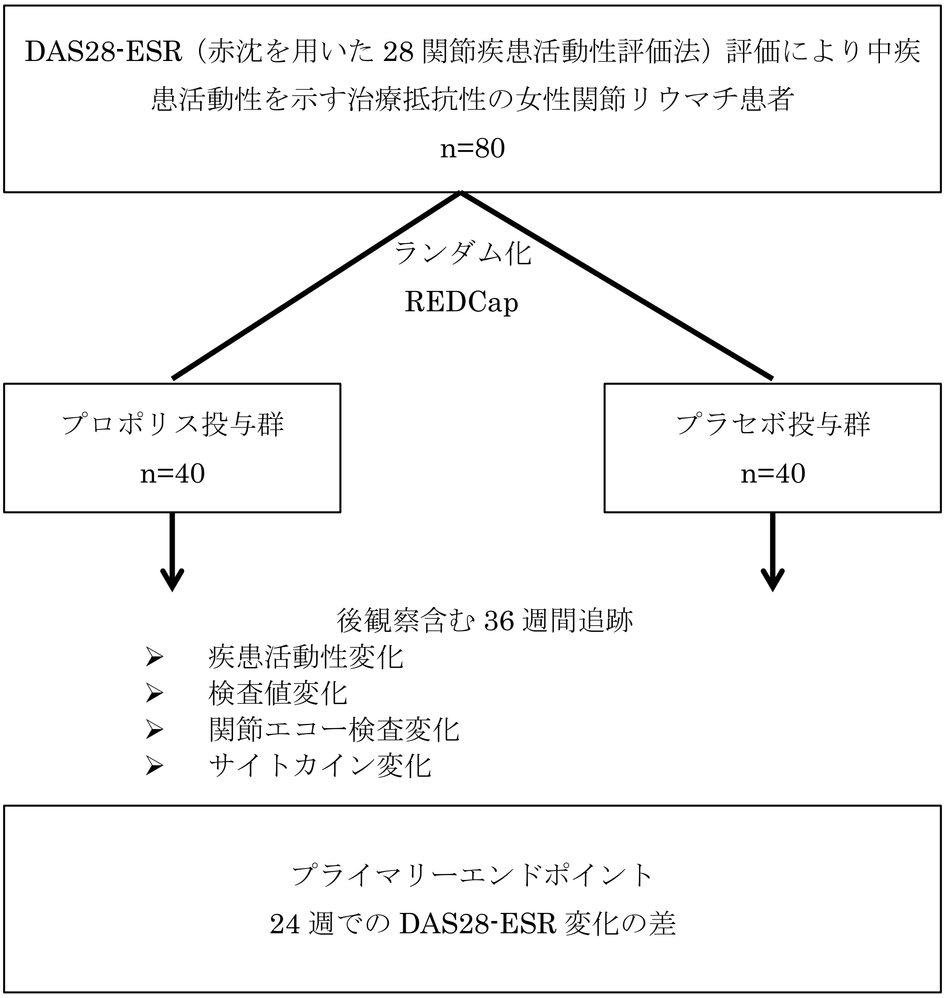

0.2　目的

関節リウマチ(RA)患者に、セイヨウミツバチが木の芽や樹液などの植物源から集めた樹脂製混合物プロポリスを摂取させることにより、RA疾患活動性が抑制されるか否かを検討する。

0.3　対象

2010年ACR/EULAR分類基準を満たし、DAS28-ESR評価で中疾患活動性を呈し、3ヶ月間治療内容に変化のない女性RA患者80名。

0.4　試験方法

DAS28-ESRによる中等度疾患活動性を呈する女性RA患者80名をプロポリス投与群(P群)40名とプラセボ投与群(C群)40名にランダムに群分けする。P群はブラジル産プロポリス含有試験球を1日5球(プロポリスとして508.5mg)摂取し、C群はブラジル産プロポリス非含有プラセボ球を1日5球摂取する。両群において介入時と24週間後のDAS28-ESRを比較検討する。その後、12週間後観察期間を設けて追跡調査を行う。

0.5　目標登録症例数と試験期間

予定登録患者数：80 名

登録期間：倫理審査委員会承認後～2019 年 3 月 31 日

試験期間：倫理審査委員会承認後～2019 年 12 月 31 日

総研究期間：倫理審査委員会承認後～2020 年 6 月 30 日

用語および略語の定義一覧

本試験実施計画書で使用する用語

用語	定義
TOMORROW 研究	Total Management of Risk Factors in Rheumatoid Arthritis Patients to Lower Morbidity and Mortality（関節リウマチ 患者の罹患率と死亡率を下げるためのリスク因子のトータルマネージメント）研究

本試験実施計画書で定義した略語

略語	定義
P 群	ブラジル産プロポリス含有試験球投与群
C 群	ブラジル産プロポリス非含有試験食プラセボ球投与群

一般項目の略語一覧

略語	省略していない表現（日本語）
RA	rheumatoid arthritis （関節リウマチ）
DAS	disease activity score （疾患活動性スコア）
ESR	erythrocyte sedimentation rate（赤血球沈降速度）
ACR	American College of Rheumatology（アメリカリウマチ学会）
EULAR	European League against Rheumatism（欧州リウマチ学会）
IL	interleukin（インターロイキン）
QOL	quality of life（生活の質）
MTX	methotrexate(メトトレキサート）
DMARDs	disease modifying anti-rheumatic drugs(疾患修飾性抗リウマチ薬)
bDMARDs	biological DMARDs(生物学的疾患修飾性抗リウマチ薬)
csDMARDs	conventional synthetic DMARDs（従来型疾患修飾性抗リウマチ薬)
TNF-α	tumor necrosis factor-alpha(腫瘍壊死因子α）
ADL	activities of daily living(日常生活動作)
CRP	c- reactive protein(C 蛋白反応性蛋白)
SDAI	Simplified disease activity index(単純化疾患活動性指標)
CDAI	Clinical disease activity index(臨床的疾患活動性指標)
mHAQ	Modified health assessment questionnaire(改変健康評価質問表)
WBC	白血球
RBC	赤血球
Hb	ヘモグロビン
Plt	血小板

AST	アスパラギン酸アミノトランスフェラーゼ
ALT	アラニンアミノトランスフェラーゼ
γ-GTP	γ グルタミルトランスペプチダーゼ
T-Bil	総ビリルビン
Cre	クレアチニン
BUN	尿素窒素
RF	リウマトイド因子
Alb	アルブミン
T-chol	総コレステロール
HDL-chol	高比重リポタンパク質コレステロール
TG	中性脂肪
Na	ナトリウム
Cl	クロール
K	カリウム
Mg	マグネシウム
P	リン
Ca	カルシウム
FBS	空腹時血糖
CCP	cyclic citrullinated peptide（環状シトルリン化ペプチド）
MMP	matrix metalloproteinase（マトリックスメタロプロテイナーゼ）
SF-36	36-Item Short-Form Health Survey
SJC	swollen joints count（腫脹関節数）
TJC	tender joints count（圧痛関節数）
GH	global health assessment（患者による全般的評価）
VAS	visual analog scale(視覚的アナログ尺度)
NSAID	non-Steroidal Anti- Inflammatory Drug（非ステロイド性抗炎症薬）
BMI	body mass index（体格指数）体重(Kg)÷（身長(m)）^{2}
REDCap	research electronic data capture
CRC	clinical research coordinator（臨床研究コーディネ ーター）

目次

0　概要 1
0.1　シェーマ 1
0.2　目的 1
0.3　対象 1
0.4　試験方法 1
0.5　目標登録症例数と試験期間 2
略語定義一覧 iii, iv
目次 v, vi, vii
1　試験の背景 1
2　試験の目的 1
3　試験食品の概要 2
3.1　試験食品 2
3.2　予期される有害反応および医療機器の場合は予期される不具合 2
4　対象疾患の診断基準と病期・病型分類 2
5　対象患者 4
5.1　選択基準 4
5.2　除外基準 5
6　症例登録・割付 5
7　試験計画 6
7.1　試験の種類・デザイン 6
7.2　試験のアウトライン 6
7.3　被験者の試験参加期間 6
7.4　試験食品の用法・用量、投与期間 6
7.5　併用薬（療法）に関する規定 7
7.6　減量・休薬の方法（試験薬調節方法） 7
7.7　試験食品の管理・交付手順 7
7.8　摂取指導情報 7
7.9　試験終了後の対応（または「後治療」） 8
7.10　試料等の保存および他の機関等の試料等の利用 8
8　観察および検査項目とスケジュール 8
9　個々の被験者の中止基準 10
10　有害事象発生時の取扱い 10
10.1　有害事象発生時の被験者への対応 10
10.2　重篤な有害事象の報告 11
10.3　その他の有害事象 11

11　　試験の終了、中止、中断 11
11.1　　試験の終了 11
11.2　　試験の中止、中断 11
12　　評価項目 11
12.1　　主要評価項目 11
12.2　　副次的評価項目 12
13　　データの集計 12
14　　統計解析 12
14.1　　データの取り扱い 12
14.2　　有効性解析 13
14.3　　副次解析 13
14.4　　中間解析 13
15　　目標症例数および設定根拠 13
15.1　　目標症例数 13
15.2　　設定根拠 14
16　　試験実施期間 14
17　　倫理的事項 14
17.1　　指針及びヘルシンキ宣言への対応 14
17.2　　倫理委員会の承認 14
17.3　　同意・説明文書及び被験者への情報提供 14
17.4　　人権への配慮（個人情報の保護） 15
17.5　　安全性・不利益への配慮 15
17.6　　相談等の対応 16
18　　試験の費用負担 16
18.1　　試験資金および利益相反 16
18.2　　患者の費用負担 16
18.3　　健康被害の補償および保険への加入 16
19　　記録の保存 16
20　　試験計画の登録および試験結果の公表 17
21　　試験組織 17
21.1　　研究代表者 17
21.2　　実施施設・試験責任医師 17
21.3　　試験事務局 17
21.4　　試験調整事務局 17
21.5　　効果安全性評価委員会 17
21.6　　モニタリング担当者（モニター） 17
21.7　　データセンター 17
21.8　　生物統計家 17

10
21.9　臨床検査受託機関 18
22　実施計画書からの逸脱または変更 18
23　モニタリング／データの品質管理 18
24　監査 18
25　参考文献 18
26　添付資料／添付図表 18

1　試験の背景

関節リウマチ(Rheumatoid Arthritis: RA)は真の原因が不明の自己免疫疾患であり、何らかの遺伝的背景に加えて喫煙や歯周病などの環境因子に晒されることにより自己免疫反応がはじまると考えられている。自己免疫反応が始まると、関節あるいは全身性に炎症が生じることで、関節の腫脹や疼痛が生じ、関節の破壊や変形に進展する。また、RAでは、炎症性サイトカインによって破骨細胞が活性化されて骨破壊が生じる。成人のRA有病率は、全世界で約0.5～1%、30～50歳代の女性に多く発症し、日本でも70万人以上の患者がいると言われている。RA患者がこのように多数存在するにもかかわらず、RAの詳細な発症原因は不明のままで、根本的な治療戦略が存在しないのが現状である。現在、RAの症状である関節の疼痛、腫脹、炎症などの疾患活動性に対する主な治療法には、薬物療法と外科的治療法があるが、薬物療法がRAの治療の基本となっている。薬物療法には、メトトレキサート(MTX)に代表される疾患修飾性抗リウマチ薬(disease modifying anti-rheumatic drugs; DMARDs)、TNF-α (tumor necrosis factor-alpha)に対するモノクローナル抗体であるインフリキシマブやIL-6 (interleukin-6)受容体抗体であるトシリズマブなどの炎症性サイトカインを分子標的とする生物学的製剤、炎症の抑制を目的とする副腎皮質ステロイド剤などが存在する。

薬物療法の問題点としては、当然種々の副作用が存在すること、生物学的製剤などは高価であり全患者に使用できるわけではないこと、全員に同様の効果を示すとは限らないこと、などが挙げられる。したがって、現在の治療目標である寛解に到達することなく継続的に治療を受けている患者も多い。

本試験で使用する健康食品は、株式会社山田養蜂場のブラジル産プロポリス含有試験食である。プロポリスはミツバチが植物の芽や樹液、花粉を集め、自らが分泌する唾液や蜜蝋を混合して作る粘着性の樹脂状物質で、巣材として用いている。山田養蜂場ブラジル産プロポリス含有試験食の抽出元のプロポリス原塊は、ブラジルのミナス・ジェライス州で採取された Baccharis dracunculifolia を主な起源植物とする。ブラジル産プロポリスは、関節炎に対しては、マウス関節リウマチモデルにおいて、疾患活動性の抑制に寄与し、その機序としてブラジル産プロポリスが、ナイーブヘルパーT 細胞が IL-17 産生ヘルパーT 細胞（Th17 細胞）に分化する過程に必要な STAT3（signal transducer and activator of transcription 3）のリン酸化を抑制し、IL-17 産生を抑制することが報告されている。以上のことから、プロポリスが RA の疾患活動性抑制に寄与する可能性があり、新たな RA 治療薬の開発につながる可能性がある。しかしながら、現時点ではマウス関節リウマチモデルにおける動物実験での研究成果しかなく、ヒトを対象としたプロポリスの疾患活動性抑制効果については不明である。そこで、本研究ではプロポリス投与により RA 患者の疾患活動性がどのように変化するかを臨床的に検証したい。疾患活動性指標には患者自身の痛みや疾患活動性に対する評価が含まれるため、盲検化が必須であり、プロポリス非含有試験球も準備し、二重盲検ランダム化試験として実施したい。

2　試験の目的

対象疾患患者：2010年ACR/EULAR分類基準を満たした女性RA患者80名。

試験食品：ブラジル産プロポリス含有試験球

評価項目：

・身体計測：身長、体重、腹囲

・疾患活動性：DAS28-ESR・DAS28-CRP・SDAI・CDAI

・ADL：mHAQ

・採血：通常検査（WBC、RBC、Hb、Ht、Plt、AST、ALT、γ-GTP、T-Bil、Cre、BUN、CRP、ESR、RF、TP、Alb、

T-chol、HDL-chol、TG、Na、Cl、K、Mg、P、Ca、FBS)、特殊検査 (抗CCP抗体、MMP-3、IL-6、IL-17、IL-10)

・血圧

・生活習慣調査：喫煙の有無、飲酒の有無、運動習慣の有無、睡眠時間、サプリメント使用の有無と種類

・ QOL:SF－36

・ 客観的関節炎評価：関節エコー検査

DAS28-ESRによる中等度疾患活動性を呈する女性RA患者80名をブラジル産プロポリス投与群(P群)40名とプラセボ球投与群(C群)40名に群分けする。その際には、施設を層に入れた、層別置換ブロック法による二重盲検無作為化による割付を行う。試験食品は株式会社山田養蜂場にて、各群40名に予備の5名分ずつを加えて、1日5球× (84日+予備7日) ×45名= 2,0475球を3ヶ月分として製造する。これが1セットで6ヶ月分2セット作成予定。P群はブラジル産プロポリス含有試験食を1日5球(Pとして508.5mg)摂取し、C群はブラジル産プロポリス非含有試験食プラセボ球を1日5球摂取する。両群において0・12・24・36週にDAS28-ESRを比較するとともに、上記項目に関しても比較検討を行う。

本試験によりブラジル産プロポリスがマウスRAモデルのみならずRA患者においても疾患活動性抑制効果を有するかを明らかとすることを目的とする。

3 試験食品の概要

3.1 試験食品

詳細は添付の試験食品概要書を参照

3.2 予期される有害反応および医療機器の場合は予期される不具合

詳細は添付の試験食品概要書を参照

4 対象疾患の診断基準と病期・病型分類

RA の診断は 2010 年 ACR/EULAR 分類基準を用い、下記のとおりとする。

ACR/EULAR 関節リウマチ分類基準 2010

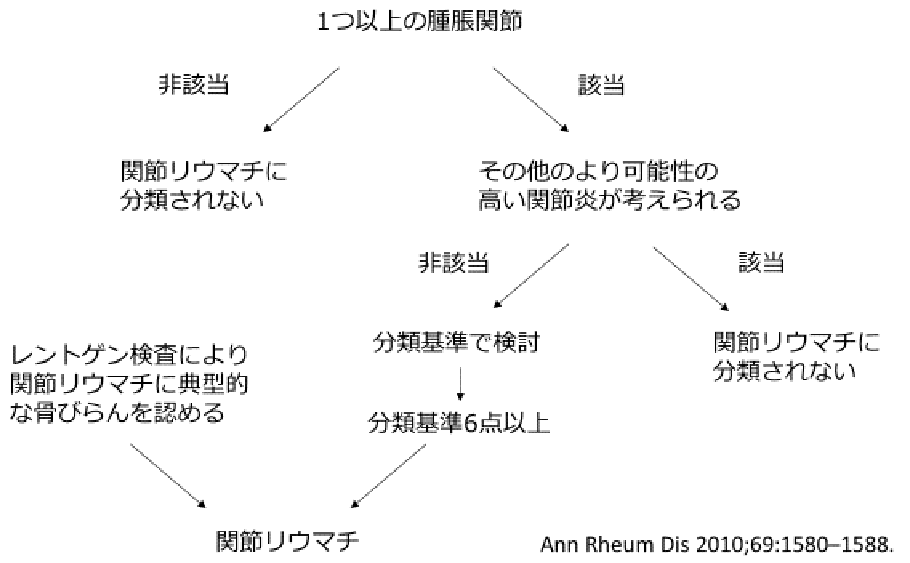

ACR/EULAR 関節リウマチ分類基準 2010

腫脹または圧痛を認める関節数	点	
・大関節1か所	0	大関節; 肩、肘、股関節、膝、足首
・大関節2-10か所	1	小関節; MCP, PIP, 2-5MTP,第1IP, 手首
・小関節1-3か所	2	
・小関節4-10か所	3	
・小関節1か所以上かつ11か所以上	5	
血清反応		
・RFとACPA両方が陰性	0	低値陽性; 正常上限以上～正常上限の3倍未満
・RFあるいはACPAが低値陽性	2	高値陽性;正常上限の3倍以上
・RFあるいはACPAが低値陽性	3	
急性反応		
・CRPとESRが正常	0	
・CRPあるいはESRが異常	1	
症状のある期間		
・6週間未満	0	
・6週間以上	1	

RA の DAS28-ESR による疾患活動性分類は下記のとおりとする。

DAS28-ESR

2.6 未満：寛解

2.6 以上、3.2 以下：低疾患活動性

3.2 より大、5.1 以下：中等度疾患活動性

5.1 より大：高度疾患活動性

DAS28-ESR = 0.56 × $\sqrt{28TJC}$ + 0.28 × $\sqrt{28SJC}$ + 0.76In(ESR) + 0.014 × GH-VAS

全身 28 関節の腫脹関節数(swollen joints count: SJC)、疼痛関節数(tender joints count: TJC)、VAS(visual analog scale)を用いた患者の一般的な状態(global health: GH)の評価、CRP (mg/dl)

5　対象患者

介入時の疾患活動性が DAS28-ESR において中等度疾患活動性（3.2 より大 5.1 以下）の女性 RA 患者。対象設定基準は、RA の治療目標が最低限低疾患活動性以下（DAS-CRP3.2 以下）であることから、まずそれ以上の疾患活動性であること、さらに高疾患活動性(5.1 より大)の場合にはより強力な薬学的治療法が選択されるべきと考え、中等度疾患活動性患者を対象とした。

5.1　選択基準

次の条件をすべて満たす患者を対象として選択する。

1)　2010年ACR/EULAR分類基準を満たしたRA患者（罹病期間は問わない）
2)　年齢：同意取得時において年齢が30歳以上70歳未満の患者
3)　性別：女性
4)　中疾患活動性である（DAS28-ESRが3.2より大、5.1以下）
5)　本試験の参加にあたり十分な説明を受けた後、十分な理解の上、患者本人の自由意思による文書同意が得られた患者
6)　生物学的製剤(bDMARDs)および抗リウマチ薬(csDMARDs)の投与が介入前12週間以上一定である
7)　NSAIDおよびステロイド（プレドニゾロン換算10 mg/日以下）投与量も同意取得前12週間以上一定である
8)　外来患者

［設定根拠］

1）RA患者を対象とした研究であり、罹病期間は研究結果に影響を与えないと考えられるため。

2）RAの疫学的背景による。外来通院患者では年齢層として30歳以上70歳未満が多いため。

3）性別の影響を受けないように割合の圧倒的に多い女性に限定した。

4）低疾患活動性以下であれば治療目標に到達しており、高疾患活動性は健康食品の対象ではないと考えるため。

5）倫理的観点による・

6)-7）RAの薬剤治療を行っている患者を対象とするため、薬剤の使用状況が疾患活動性に強く影響するため、影響を除外するため。

8）3か月に1回疾患評価を行うため。

5.2　除外基準

以下のいずれかに抵触する患者は対象から除外する。

1)　定期的に検査に来られない患者

2)　分類不能関節炎患者

3)　同意取得以前4週間以内にサプリメントとしてプロポリスを摂取している患者

4)　著しい肥満がある患者 (BMI30以上)

5)　肝機能に異常を有する患者 (ASTもしくはALTが正常上限値の3倍以上)

6)　腎機能に異常を有する患者 (BUN 25 mg/dL 以上もしくは血清クレアチニン2.0 mg/dL以上)

7)　妊娠中あるいは授乳中の患者

8)　食品アレルギー歴を有する患者

9)　その他、試験責任(分担)医師が被験者として不適当と判断した患者

[設定根拠]

1) 有効性評価への影響および安全性への配慮のため

2) RA患者への有効性評価のため

3)-4) 有効性評価への影響のため

5)-9) 安全性への配慮のため

6　症例登録・割付

試験食品の割付・コード化：二重盲検化

試験食品およびプラセボ食品は山田養蜂場にて製造後に、みつばち健康科学研究所において 1 日分の 5 球を小袋に入れ、84+予備 7 日分の 91 袋を 1 名 3 ヶ月分としてセット組みする。その上で、みつばち健康科学研究所において、全セット（1 群 40 名+予備 5 名×2 群=90 名）にランダムに試験食品割付コードを割り当て、割り当て表を REDCap ランダム化担当者にのみ送付する。みつばち健康科学研究所においては、1)試験食品の識別不能性を確保するため全試験球を白色コーティングする。2)試験食品割付コード表は、開鍵時までみつばち健康科学研究所および REDCap 試験食品割付・コード化担当者が保管し、他の者には開示しない。開鍵は、緊急避難的に該当症例について行う場合を除いて、試験が終了し各症例のデータが固定するまで行わない。なお、試験食品は、1 症例分を 1 単位(1 セット)とする。

被験者の登録方法：ランダム化

試験責任医師あるいは試験分担医師は、被験者登録に先立って、各医療機関の承認が得られた説明文書(付表の説明文書または各医療機関で改変を加えた説明文書) を被験者となるべき者に渡し、原則として「人を対象とする医学系研究に関する倫理指針」で定める内容を口頭で詳しく説明の上、試験参加について自由意思による同意を文書にて得る。同意取得に際しては、被験者となるべき者に試験に参加するか否かを判断するのに十分な時間と質問する機会を設け、質問に十分に答える。

同意取得後、必要項目をREDCapシステム内に構築された被検者登録画面より入力し適格性確認を実施する。適格性が確認された被検者について同じくREDCapシステム内の試験食品割付画面より入力を行い、無作為割付を実施する。なお試験食の配布は同日行うこととする。

登録システム:REDCapシステム
URL：ocu.jp/redcap 24時間受付可能

7　試験計画

7.1　試験の種類・デザイン

試験デザインは、プラセボを対照とする多施設二重盲検ランダム化並行群間比較試験である。食品の効果を検証する際に最もエビデンスレベルの高い方法として知られている。

7.2　試験のアウトライン

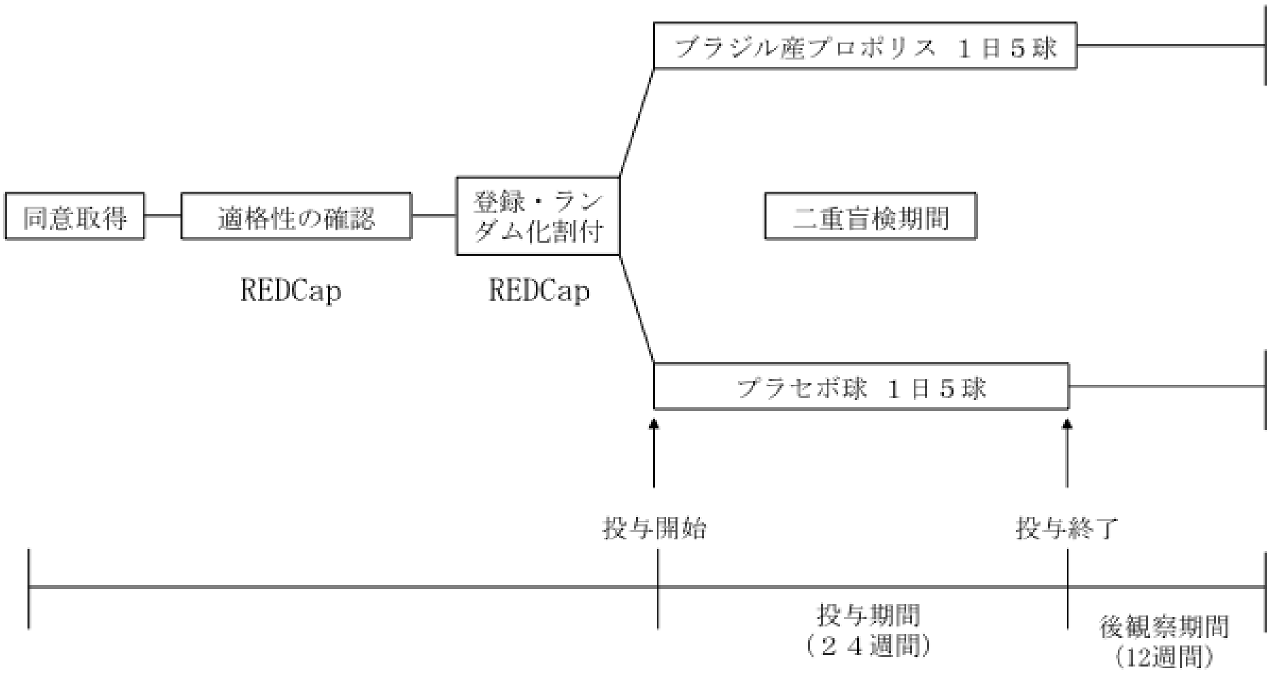

7.3　被験者の試験参加期間

全症例最大40週追跡時まで。

7.4　試験食品の用法・用量、投与期間

投与量は、P群はブラジル産プロポリス含有試験球を連日1日5球、C群はプラセボ球を連日1日5球投与する。服用時間の指定はなし。5球ごとの小袋包装で試験食品を提供し、一度に5球を摂取することを念頭に置いているが、試験参加者が分割して摂取することは許容する。1日に5球を摂取しなかった場合は翌日には服用せずに次回来院時に袋ごと返却する。1日5球以上の摂取は禁止する。訪問ごとに小袋ごと回収するので、飲み残しは、この時点で確認し記録する。投与期間は24週間とする。

【投与量及び投与期間の設定根拠】

本試験で用いるブラジル産プロポリス含有試験食の用量は、株式会社山田養蜂場が目安量として設定している１日 4-6 球の中間の５球(ブラジル産プロポリス抽出物粉末 508.5mg)とした。

7.5　併用薬（療法）に関する規定

１）併用薬（療法）：試験24週間は可能な限り併用薬の変更は行わない。ただし、疾患活動性が充分にコントロールできない場合には、その限りではない。生物学的製剤も介入12週間前より一定の投与であれば併用可能。

２）併用禁止薬（療法）：プロポリスの摂取（自費購入）

３）併用制限薬（療法）：記載事項なし

４）併用注意薬（療法）：ワーファリンカリウム

５）併用可能薬（療法）：記載事項なし

7.6　減量・休薬の方法（試験薬調節方法）

本試験では食品を扱うため減量・休薬の方法は設けない

7.7　試験食品の管理・交付手順

各施設に予備を含めて試験食品およびプラセボを１：１の割合になるようにセット組みして、山田養蜂場ミツバチ健康科学研究所より施設に送付する（大阪市立大学 26 名分、白浜はまゆう病院および北出病院は各 32 名分で北出病院分は白浜はまゆう病院へ送付)。いずれも目標症例数よりも多いため、不足は生じないと考える。ブラジル産プロポリス球およびプラセボ球については CRC にて管理を行う。ランダム化・割付後に投与開始時と 12 週の時点でそれぞれ 12 週分を症例毎に手渡しする。

保管場所：治験薬管理室(大阪市立大学分)および白浜医療福祉財団骨リウマチ疾患探索研究所（白浜はまゆう病院、北出病院分)

保管責任者：試験責任医師小池達也

7.8　摂取指導情報

- 摂取時刻：問わない
- 摂取方法：1回1-5球を噛まずに水と共に服用する
- 飲み忘れたときの指導方法：飲み忘れた際はその日の分はスキップし、翌日以降通常通りの服用とする
- 保管方法：室温で保存
- 残ったサンプル・容器の返却方法：12 週、24 週の外来診察時に持参

7.9　試験終了後の対応（または「後治療」）

被験者が試験終了後においても試験の結果により得られた最善の予防、診断および治療を受けることができるよう努める。

7.10 試料等の保存および他の機関等の試料等の利用

- 保存：サイトカイン測定のための血清を大阪市立大学および骨リウマチ疾患探索研究所にて測定まで-80℃で保存する。
- 使用方法：株式会社ＬＳＩメディエンスにて全サンプル回収後に一括測定する。
- 保存期間：測定データ確定まで
- 管理者:試験責任者小池達也
- 廃棄方法: データ確定後、匿名化した上で廃棄する
- 研究者等が情報等を正確なものにするよう指導・管理し、人体から取得された試料及び情報等の漏えい、混交、盗難、紛失等が起こらないよう必要な管理を行う
- 人体から取得された試料及び情報等の保管に関する手順書に従い、管理の状況について病院長に報告する

8　観察および検査項目とスケジュール

スケジュール表に従い、以下の調査を実施する。

(1) 患者背景

カルテ番号、患者氏名、性別、生年月日、合併症、既往歴、現病歴、前治療、併用薬（併用療法・現治療内容)、生活習慣(喫煙の有無、飲酒の有無、運動習慣の有無、睡眠時間、サプリメント使用の有無と種類)。ただし、カルテ番号や患者氏名はREDCap（症例報告書）には入力しない。

(2) 試験食品の摂取状況の確認

当該調査期間における摂取状況を％で評価し、REDCapに記録する。被験者は各visitで、未使用の包装材を含むすべてのサンプルを実施医療機関に返却する。CRCは配布球数と返却球数から摂取率を計算する。症例に対しては、来院ごとに前回来院日翌日から来院当日までの試験食品の摂取状況を調査のうえ、摂取遵守を依頼する。

(3) 自覚症状・他覚所見の確認,疾患活動性評価

質問票および触診並びに検査値より以下の計算式に従って活動性を数値化する。

$DAS28\text{-}ESR = 0.56 \times \sqrt{28TJC} + 0.28 \times \sqrt{28SJC} + 0.76\mathrm{In}(ESR) + 0.014 \times GH\text{-}VAS$

$DAS28\text{-}CRP = 0.56 \times \sqrt{28TJC} + 0.28 \times \sqrt{28SJC} + 0.36\mathrm{In}(CRP \times 10 + 1) + 0.014 \times GH\text{-}VAS + 0.96$

SDAI = 28TJC + 28SJC + PtGH-VAS(患者) + DrGH-VAS(医師) + CRP (mg/dl)

CDAI = 28TJC + 28SJC + PtGH-VAS(患者) + DrGH-VAS(医師)

(4) 有害事象と副作用の確認：各種検査値異常も含め内容、発現時期・消失時期、程度、処置、転帰、重篤性評価、試験食品との関連性等をカルテおよび症例報告書に記載する。必要があれば追跡調査する。程度については 1)軽度：無処置で投与継続可能な状態、2)中等度：何らかの処置により投与継続可能な状態、3)重度：投与を中止あるいは中止すべき状態とする。

(5) 身長、体重、腹囲

(6) 血圧・脈拍数

(7) 血液学的検査

WBC(白血球数)、RBC(赤血球数)、Hb(ヘモグロビン)、Ht(ヘマトクリット)、Plt(血小板)

(8) 血液生化学検査

AST、ALT、γ-GTP、T-Bil(総ビリルビン)、Cre(クレアチニン)、BUN(尿素窒素)、CRP、ESR(赤血球沈降速度)、RF(リウマトイド因子)、TP(総タンパク)、Alb(アルブミン)、T-chol(総コレステロール)、HDL-chol、TG(中性脂肪)、Na、Cl、K、Mg、P、Ca、FBS(空腹時血糖)

(9) その他の特殊検査

・抗 CCP 抗体、MMP-3、IL-6、IL-17、IL-10

外注会社名：株式会社ＬＳＩメディエンス

・QOL：SF－36

・ADL：mHAQ

・ 疾患活動性：DAS28-ESR・DAS28-CRP・SDAI・CDAI

・ 関節エコー検査：症状の強い側（観察期間中変更なし）の手関節・第2-3中手基節間関節・第2-3中枢指節骨間関節・第2および5中足基節間関節の7関節のグレースケール（0-3）およびパワードップラースケール（0-3）

スケジュール表

項　目	前観察期間	投与開始日	投与期間	投与終了日	後観察期間
時　期	1-4 週前	0 週	投与 12 週後<br>(±7 日)	投与 24 週後<br>(±7 日)	終了(中止)<br>12 週後
受　診	受診 1	受診 2	受診 3	受診 4	受診 5
同意取得	○				
患者背景の確認	○				
試験食品投与		←	――	→	
自覚症状・他覚所見	○	○	●	●	●
有害事象の観察[a]			●	●	●
身体計測[b]		○		●	
血圧(座)・脈拍測定		○	●	●	●
血液学的検査		○	●	●	●
血液生化学検査		○	●	●	●
関節エコー検査		○	●	●	●
QOL 評価		○		●	
疾患活動性評価[c]	○	○	●	●	●

○印は試験食品投与開始前に行う項目、●印は試験食品投与開始後に行う項目

a:有害事象は、副作用など好ましくないすべての事象のことで、試験食品との因果関係は問わない。
b:身体計測として身長、体重、腹囲を測定する。
c:疾患活動性評価として DAS28-ESR,DAS28-CRP,SDAI,CDAI を評価する
血液検査は食事の影響を除外するため絶食条件下で行う

9　個々の被験者の中止基準

試験責任医師または試験分担医師は、以下の理由によって試験の継続が困難となった被験者について試験を中止し、被験者の利益性に基づき適切に処置する。また、予定されている調査／検査を可能な限り速やかに実施し、判定を行う。(同意撤回などによって実施できない場合を除く。) 試験食品投与開始後に同意の撤回があった場合は、試験食品の効果不発揮あるいは有害事象によるものか、あるいは偶発的事象（転居など）によるものかをできるだけ明らかにし、有効性・安全性評価の対象となる症例としての採否の参考となるように記録する。

試験責任医師または試験分担医師は、中止日、中止理由、コメントを症例報告書に記録する。

中止後の対応として、下記 4)-6),10) の項目に該当した場合は、安全性確認のため血液学的検査、血液生化学検査を実施する。また、各症状に対して医師が必要と判断した検査を実施する。それぞれ検査結果に応じて医師が必要と判断した処置・治療を実施し、1 年間追跡調査を実施する。特に、有害事象発生により中止した場合は、可能なかぎり原状に回復するまでフォローする。

1)　被験者から試験参加の辞退の申し出や同意の撤回があった場合
2)　その他被験者側の理由（多忙、転院、転居など）
3)　登録後に適格性を満足しないことが判明した場合
4)　原疾患の悪化のため、試験食品の投与継続が好ましくないと判断された場合
5)　合併症の増悪により試験の継続が困難な場合
6)　有害事象により試験の継続が困難な場合
7)　妊娠が判明した場合
8)　試験全体が中止された場合
9)　その他の理由により、医師が試験を中止することが適当と判断した場合

10　有害事象発生時の取扱い

10.1　有害事象発生時の被験者への対応

「有害事象」とは、試験食品が投与された時に起こるあらゆる好ましくない、あるいは意図しない徴候、症状または病気とし、試験食品との因果関係の有無は問わない。

試験責任医師または試験分担医師は、有害事象を認めたときは、直ちに適切な処置を行うとともに、カルテならびに症例報告書に齟齬なく記載する。また、試験食品の投与を中止した場合や、有害事象に対する治療が必要となった場合には、被験者にその旨を伝える。

緊急避難的に試験食品の識別を行う必要がある場合は、研究代表者等を通じて試験食品割付・コード化担当者に依頼し、当該症例について開封結果の開示を受ける。

重篤な有害事象の発生を認めたときは、当該研究との因果関係の有無にかかわらず、全ての重篤な有害

事象を速やかに病院長に報告する

10.2 重篤な有害事象の報告

「重篤な有害事象」とは、投与量に関わらず、あらゆる好ましくない医療上の出来事のうち、以下の条件に該当するものをいう。

(1)死に至るもの

(2)生命を脅かすもの

(3)治療のため入院が必要となるもの

(4)永続的又は顕著な障害・機能不全に陥るもの

(5)子孫に先天異常を来すもの

試験責任医師は、侵襲を伴う研究を実施している間に重篤な有害事象の発生を認めたときは、当該研究との因果関係の有無にかかわらず、全ての重篤な有害事象を速やかに病院長（大阪市市立大学においては治験審査委員会）に報告するとともに、多施設共同試験において試験と重篤な有害事象の因果関係を否定できない場合は、他の医療機関の責任医師に報告する。

10.3 その他の有害事象

その他の有害事象については、「8.観察および検査項目(4)有害事象と副作用の確認」に記載した手順により、症例報告書に記載する。

11 試験の終了、中止、中断

11.1 試験の終了

各施設での試験の終了時には、試験責任医師は、速やかに試験終了報告書を病院長に提出する。多施設において実施する場合は、研究代表者にも終了報告書を提出する。

11.2 試験の中止、中断

試験責任医師は、以下の事項に該当する場合は試験実施継続の可否を検討する。

1)試験食品の品質、安全性、有効性に関する重大な情報が得られたとき。

2)被験者のリクルートが困難で予定症例を達成することが到底困難であると判断されたとき。

3)審査委員会により、実施計画等の変更の指示があり、これを受入れることが困難と判断されたとき。

なお、審査委員会により、中止の勧告あるいは指示があった場合は、試験を中止する。多施設で行う場合は、研究代表者あるいは試験計画書で規定する委員会等で上記の事項を検討し、試験の継続の可否を検討する。試験の中止または中断を決定した時は、速やかに病院長にその理由とともに文書で報告する。

12 評価項目

12.1 主要評価項目

試験食品摂取開始から24週時点での疾患活動性 DAS28-ESR の変化量を本試験における主要評価項目とする。

【設定根拠】

本試験では RA の疾患活動性に対するブラジル産プロポリスの抑制効果を検討する。上記疾患活動性の項目については ACR/EULAR のみならず世界中での RA の疾患活動性を評価する際のゴールドスタンダードである。

12.2 副次的評価項目

1） 摂取開始後 12 週、24 週、36 週の DAS28-CRP、SDAI、CDAI
2） 摂取開始後 24 週の QOL（SF-36）
3） 摂取開始後 12 週、24 週、36 週の ADL：mHAQ
4） 摂取開始後 12 週、24 週、36 週の関節エコー所見（スケール変化）
5） 摂取開始後 24 週までの試験食品の中断・中止等を要した有害事象
6） 摂取開始後 36 週までの重篤な有害事象
7） 摂取開始後 36 週までのすべての有害事象

【設定根拠】

1） DAS28-ESR以外の疾患活動性評価方法で、それぞれ一長一短があるため、他の疾患活動性評価も実施する。
2）対象者の一般的QOL評価ツールとして、SF-36がその簡便性や信頼性から数多くの論文で報告されている。疾患活動性抑制に伴うQOLの改善効果を評価する。
3）ACR/EULARのみならず世界中でRA患者のADL評価をする際のゴールドスタンダードである。疾患活動性抑制に伴いADLの改善効果を評価する。
4）疾患活動性評価には主観的項目が多数含まれるために、より客観的評価を得る。
5-7）試験食品の安全性評価のため。

13 データの集計

すべてのデータは REDCap システム内に構築された eCRF（電子症例報告書）へ必要項目を入力しデータを収集する。なお各 visit の入力は原則、被験者来院日より 7 日以内に入力する。カルテ番号などの個人情報は入力項目がないため、個人を特定できる対応表は別に作成し、責任医師および分担医師が厳重に保管する。

14 統計解析

14.1 データの取り扱い

計画書に規定された観察・検査項目のデータについて、時期別の集計を行う際には、許容範囲から外れた日もしくは時間に実施した検査項目のデータは欠損扱いとする。

欠損値に対し、主解析を行う際には LOCF により値を補完する。

14.2 有効性解析

解析対象集団は最大の解析対象集団（full analysis set; FAS）と設定し、本試験に割付けされ、一度でも試験食品を摂取しかつ試験食品投与後に一度でも有効性の評価が実施された被験者と定義する。また、実施計画書に適合した対象集団（per protocol set; PPS）による解析も補足的に行う。仮説検定には5%の有意水準で両側検定、両側95%信頼区間を用いる。

主要評価項目について介入終了時の DAS28-ESR を目的変数、群(対照、介入)を説明変数、0 週時の DAS28-ESR の値を共変量としたモデルを用いて、群の係数についての有意性検定を行う。なお、LOCF による欠損値補完に伴う結果の頑健性確認のため、主要評価項目に関し、混合効果モデルによる解析を補完的に行う。

その際には介入終了時の DAS28-ESR の正規性をヒストグラムにより確認し、正規性が見られない場合は正規分布に従うように log 変換等の変数変換を施した下で解析に用いる。また、副次的な解析として、目的変数を DAS28-ESR としたときに、群(対照、介入)と時点(12 週、24 週)と群と時点の交互作用を説明変数とし、0 週時の DAS28-ESR の値を共変量、被験者を変量効果とした混合効果モデルを用いて解析を行い、時点の相関構造は無構造とする。

14.3 副次解析

研究の主たる解析結果の補足的な考察を行う目的で副次評価項目に対する解析を実施する。なお副次評価項目については探索的な意味合いで解析を行うため多重性の調整は行わない。1）摂取開始後 12 週、24 週、36 週の DAS28-CRP、SDAI、CDAI、2）摂取開始後 24 週の QOL（SF-36）、3）摂取開始後 12 週、24 週、36 週の ADL：mHAQ 4）摂取開始後 12 週、24 週、36 週の関節エコー所見（スケール変化）には上記主要評価項目と同様の解析を行う。有害事象の発現の有無について、投与群ごとに頻度表を作成する。なお上記有害事象のうち、重篤な有害事象が発生した場合にはイベント発生までの生存率解析を行うため、カプランマイヤー曲線を作成し、0 週時の DAS28-ESR を共変量とした Cox 比例ハザードモデルを用いて群間比較を行う。臨床検査の定量データは、投与群ごとに時点毎の測定値及び試験食品投与開始前からの変化量について要約統計量を算出し、散布図を作成する。その他、定性データは投与群ごとに頻度表を作成する。

14.4 中間解析

投与は既に世界中で販売されている健康食品であり、重篤な有害事象が発生する可能性は低い。また逆に、強力な薬剤でもないために短期間に効果が出現する可能性も低い。全投与調査期間は 24 週であるので、中間解析を行うメリットは少ないと考え、中間解析は行わない。

15 目標症例数および設定根拠

15.1 目標症例数

参加施設数 3 施設、目標症例数 80 例

15.2　設定根拠

関節リウマチ患者を対象に本試験食品を含むプロポリスを投与した臨床試験はない。類似試験としてRA 患者に対する地中海式ダイエットによる介入効果を検証した臨床試験（Ann Rheum Dis 2003: 62: 208-214）において、対照群 25 名と介入群 26 名の 12 週後の DAS28-ESR の平均値の差は 0.4 ポイントであった(12 週時点で 2 群間に統計的有意差を認める)。一方、当該試験において、被験者適格基準に DAS28-ESR を用いておらず、試験参加者の疾患活動性のばらつきが大きいことが懸念された。本試験では臨床における疾患管理等の観点からエントリー基準を中疾患活動性に限定しており、試験食品による DAS28-ESR への介入効果は上記試験と同程度またはそれ以上期待できることから、2010 年より本邦にて研究責任者を含む研究グループにて実施中の関節リウマチ患者と健常者のコホート研究対象者のデータを参考に、以下の通り仮説を設定した。

我々が実施しているコホート研究である TOMORROW 研究に 2016 年時点で継続参加している関節リウマチ患者 182 名のうち中疾患活動性を示す RA 患者 67 名の 1 年間の標準治療下における疾患活動性の変化量平均-0.22 に加え、プロポリスによる上乗せ効果を-0.5 と仮定する。σ（予想されるアウトカムの標準偏差）= 0.78 とすると、有意水準 α=0.05 で δ（2 群間で 24 週後に DAS28-ESR の差として見たい値）= 0.5 を 2 標本 t 検定により 80%の検出力で検出するためには、各群で少なくとも 39 名が必要となる。試験参加に伴う被験者の負担等を考慮し、試験期間中の脱落はほとんどないと考え、各群 40 名、計 80 名を目標症例数とした。

16　試験実施期間

症例登録期間：　承認後　から　2019　年　3　月　31　日

（登録締切　2019 年 3 月 31 日）

症例追跡期間：　承認後　から　2019　年　12　月　31　日

17　倫理的事項

17.1　指針及びヘルシンキ宣言への対応

本試験に関係する全ての研究者ならびに研究協力者は、「ヘルシンキ宣言（2013 年ブラジル フォルタレザ改訂）（日本医師会訳）の精神を遵守し、かつ本試験実施計画書及び「人を対象とする医学系研究に関する倫理指針」（平成 26 年文部科学省・厚生労働省告示第 3 号、平成 29 年 2 月 28 日一部改正）に従って本試験を実施する。

17.2　倫理委員会の承認

本研究は大阪市立大学医学部附属病院の審査委員会、白浜はまゆう病院倫理委員会および北出病院倫理委員会の承認を得て実施する。

17.3　同意・説明文書及び被験者への情報提供

各施設の審査委員会等で承認の得られた説明文書・同意文書を患者さんに渡し、文書および口頭による十分な説明を行い、患者さんの自由意思による同意を文書で得る。

患者さんの同意に影響を及ぼすと考えられる有効性や安全性等の情報が得られたときや、患者さんの同

意に影響を及ぼすような実施計画等の変更が行われるときは、速やかに患者さんに情報提供し、試験等に参加するか否かについて患者さんの意思を予め確認するとともに、事前に各施設の審査委員会等の承認を得て説明文書・同意文書等の改訂を行い、患者さんの再同意を得る。

説明文書には以下の項目を含めるものとする。

1. はじめに：自主臨床試験について
2. あなたの病状
3. この臨床試験の目的
4. この試験の方法
5. この試験の予定参加期間
6. 予測される利益およびリスク
7. この試験に参加されない場合の治療法とその内容
8. 治療にかかる費用
9. この試験を中止させていただく場合があること
10. この試験への参加に同意された場合に守っていただくこと
11. この試験中に、あなたの健康に被害が生じた場合について
12. 研究から生じる知的財産権と利益相反について
13. この試験への参加は、あなたの自由意思によること（機器の試験においては試験の参加を取りやめる場合の機器の取扱いに関する事項を含む）
14. この試験に関する情報は、随時ご連絡します
15. この試験結果が公表される場合でも、プライバシーは守られること
16. この試験に参加された場合、あなたのカルテなどが試験中あるいは試験終了後に調査されることがあること
17. 資料（試料を含む）の保存、研究終了後の利用又は廃棄方法
18. 相談窓口

17.4　人権への配慮（個人情報の保護）

実施に際しては、被験者の人権、福祉及び安全性を最大限に確保する。試験責任医師または分担医師は、本試験の実施にあたっては倫理的な配慮を慎重にし、試験内容について十分説明した上で、患者本人の同意を文書により得る。試験に参加するか否かは、被験者本人の自由意思により決定され、同意後であっても、被験者本人の意思によりいつでも中止が可能である。また、参加中止に伴う不利益は受けない。本試験で知り得た情報は、個人が同定できる形ではいかなる状況においても公表せず、かつ厳重な管理下で保管される。また、本人が希望すれば、本人の情報は本人にのみ文書にて報告する。

17.5　安全性・不利益への配慮

有害事象発生時には速やかに適切な診察と処置を行う。それでも改善せず中等度～高度の有害事象が持続する場合は、当該被験者への試験を中止する。

17.6　相談等の対応

研究対象者又はその代諾者等及びその関係者からの相談、問合せ、苦情等に適切かつ迅速に対応する。

18　試験の費用負担

18.1　試験資金および利益相反

＜研究資金および研究組織の利益相反＞

本試験は、本研究実施分担者の松本佳也が株式会社山田養蜂場みつばち研究助成基金に応募して採択された課題に対する助成金 200 万円を研究費の一部とする（本研究費は共同研究施設である白浜医療福祉財団にて管理する)。しかし、ヒトを対象とした臨床試験であるため、医師資格を持つ小池達也（研究助成申請時より共同研究者）が研究責任者に就任し研究費も受領している。試験食品はプロポリス試験球およびプラセボを山田養蜂場から無償で提供を受ける。株式会社山田養蜂場は、試験食品に関する情報は提供するが、試験の解析に係わることはない。研究費の不足分は高齢者運動器変性疾患制御講座の自己研究費（奨学寄付金など）を当てる。

＜研究者の利益相反＞

本研究に関して、株式会社山田養蜂場みつばち研究助成基金により研究助成（試験球提供）は受けているが、本試験の研究代表者および試験分担医師には、それ以外に本研究に関して開示すべき利益相反はない。

研究代表者は、本試験の計画・実施・報告において、試験の結果および結果の解釈に影響を及ぼすような新たな「利益相反」が生じていないか研究者に継続的に（例えば 1 年に 1 度年度初めに）確認し、試験の実施が被験者の権利・利益をそこねることがないことを確認する。

18.2　患者の費用負担

本試験は全て保険診療の範囲内で実施される。保険適応外の検査は研究費用で実施する。また、被験者の通院回数、検査実施頻度も通常の診療と同程度であり、試験参加による被験者の負担増加はないと考えられる。また、参加することによる謝礼も発生しない。

18.3　健康被害の補償および保険への加入

本試験にて実施が予定される検査は特殊検査（SF-36 やサイトカイン検査）を除いて試験参加の有無によらず日常診療において同頻度で実施される検査であり、すべて保険診療の範囲内で行う。本試験に起因して健康被害が生じた場合は、通常の保険診療と同様に患者の健康保険を用いて治療を行う。その際の医療費の自己負担分の支払い、休業補償、差額ベッド料金の補填などの補償については、この研究に関わる組織・施設、および医師等の個人のいずれも被験者に対して補償は行わない。ただし、因果関係が明確で、一定水準以上の健康被害（死亡または重度障害）が生じた際には臨床研究補償保険により補償を行う。臨床研究補償保険としては、1 名 1 億円・1 事故 3 億円の補償額での契約を予定している。

19　記録の保存

本試験は、侵襲を伴う介入研究に該当するため、少なくとも、当該研究の終了について報告された日から 5 年を経過した日又は当該研究の結果の最終の公表について報告された日から 3 年を経過した日のいずれか遅

い日までの期間、適切に保管する。

20　試験計画の登録および試験結果の公表

被験者登録を開始する前に、試験計画の内容を公開登録システム UMIN に登録する。結果の公表については、適切な学術集会並びに医学雑誌にて実施する。また、結果の最終の公表を行ったときは、遅滞なく病院長へ報告する。

21　試験組織

21.1　研究代表者

小池達也　　大阪市立大学大学院医学研究科高齢者運動器変性疾患制御講座　特任教授　06-6646-6010

21.2　実施施設・試験責任医師

大阪市立大学医学部附属病院　高齢者運動器変性疾患制御講座　特任教授　小池達也　06-6646-6010

白浜はまゆう病院　　整形外科　小池達也　部長　0739-43-6200

北出病院　　リウマチ科　小池達也　非常勤医師　0738-22-2188

21.3　試験事務局

大阪市立大学医学研究科高齢者運動器変性疾患制御講座

小池達也（試験事務局代表）、中塚智子

〒545-8585　大阪市阿倍野区旭町 1-4-3

電話：06-6646-6010、FAX：06-6646-6010

21.4　試験調整事務局

特に設置しない

21.5　効果安全性評価委員会

特に設置しない

21.6　モニタリング担当者（モニター）

大阪市立大学大学院医学研究科高齢者運動器変性疾患制御講座　中塚智子

21.7　データセンター

REDCap に入力により症例報告書作成とする。REDCap 管理は高齢者運動器変性疾患制御講座特任講師杉岡優子

21.8　生物統計家

大阪市立大学大学院医学研究科医療統計学教授　新谷歩

21.9　臨床検査受託機関

株式会社ＬＳＩメディエンス

委託業務：IL-10、IL-17、IL-6、MMP-3、抗 CCP 抗体の測定

監督方法：最終エントリー者の最終採血が終了後にサンプルを渡すため、会社での保存などはなし。データの信頼性を最終的にチェック。

22　実施計画書からの逸脱または変更

実施計画書や説明文書・同意文書の改訂を行う場合は予め倫理委員会等の承認を必要とする。ただし、被験者の緊急の危険を回避するためのものであるなど医療上やむを得ないものに関する変更である場合には、この限りではない。

試験責任医師または試験分担医師は、試験実施計画書からの重大な逸脱があった場合は、逸脱事項をその理由とともに記録する。

試験責任医師は、試験食品の品質、有効性及び安全性に関する事項、その他試験を訂正に行うために重要な情報を知ったときに、必要に応じて実施計画書を改訂する。有効性試験実施計画書からの重大な逸脱があった場合は、逸脱事項をその理由とともに記録する。

23　モニタリング／データの品質管理

本試験では、研究の科学的・倫理的な質の向上と教育を目的として施設訪問モニタリングを行うことがある。モニターは、研究責任者が要求する事項について確認することにより、臨床研究が適切に実施されていること、必要な事項が正確に記録されていること及びデータの信頼性が十分に保たれていることを保証する。本試験で実施する際には試験事務局である大阪市立大学大学院医学研究科高齢者運動器変性疾患制御講座がモニタリングを担当する。

24　監査

実施予定なし。

25　参考文献

Aletaha D, Neogi T, Sikman AJ, Funovits J, Felson DT, Bingham CO, et al. 2010 Rheumatoid arthritis classifi cation criteria: an American College of Rheumatology/European League Against Rheumatism collaborative initiative. Ann Rheum Dis 69:1580-1588, 2010

Tanaka M, Okamoto Y, Fukui T, Masuzawa T.: Suppression of interleukin 17 production by Brazilian propolis in mice with collagen-induced arthritis. Inflammophamacology 20:19-26, 2012

Skoldstam L., Hagfors L., Johansson G.: An experimental study of a Mediterranean diet intervention for patients with rheumatoid arthritis. Ann Rheum Dis 62:208-214, 2003

26　添付資料／添付図表

特になし
