# Supplementary material for: Double-blinded randomized controlled trial to reveal the effects of Brazilian propolis intake on rheumatoid arthritis disease activity index; BeeDAI
Source: PLoS One. 2021 May 27;16(5):e0252357. doi: 10.1371/journal.pone.0252357 (PMC8158978; doi:10.1371/journal.pone.0252357)
Supplement: Attachment — Submitted filename: Response to Reviewers (1).docx [file pone.0252357.s009.docx]

Dear Editor and Reviewers;

We would like to thank you for your very constructive criticisms and editorial suggestions regarding our manuscript. These were very helpful in our sincere efforts to improve our manuscript. We addressed all the concerns point-by-point, **depicting Reponses by use of bold font**, and revised the manuscript accordingly. We hope that our response meets with your approval. We indicate revised wording with yellow highlighting in the text.

1. Please ensure that your manuscript meets PLOS ONE's style requirements, including those for file naming. The PLOS ONE style templates can be found at

https://journals.plos.org/plosone/s/file?id=wjVg/PLOSOne_formatting_sample_main_body.pdf and

https://journals.plos.org/plosone/s/file?id=ba62/PLOSOne_formatting_sample_title_authors_affiliations.pdf

**Response: We checked the PLOS ONE style templates and adapted the format to PLOS ONE's style.**

2. Thank you for submitting your clinical trial to PLOS ONE and for providing the name of the registry and the registration number. The information in the registry entry suggests that your trial was registered after patient recruitment began. PLOS ONE strongly encourages authors to register all trials before recruiting the first participant in a study.

**Response: Thank you for pointing out. The date of the first-patient-in for this study was July 3, 2018. In advance, we registered this trial with UMIN Clinical Trials Registry on April 7, 2018. However, Japan's Clinical Trials Act went into effect on April 1, 2018, and it was verified whether our research falls under the category of Specified Clinical Research. As a result, it was confirmed that our study applicable under the category of Specified Clinical Research, and we have again registered our study with Japan Registry of Clinical Trials (jRCT) as Specified Clinical Research. The date for this registration for jRCT was March 20, 2019, which was after recruiting the first participant. We have added a note on this point to the text.**

As per the journal’s editorial policy, please include in the Methods section of your paper:

1) your reasons for your delay in registering this study (after enrolment of participants started);

**Response: We have described the reason in the Method section.**

2) confirmation that all related trials are registered by stating: “The authors confirm that all ongoing and related trials for this drug/intervention are registered”.

**Response: We have added this statement to the Methods section.**

3. Thank you for stating the following in the Financial Disclosure section:

"YM received Yamada Research Grant

Specific grant numbers

Yamada Research Grant (grant No.249)

Full names of commercial companies that funded the study or authors

Yamada Bee Company, Inc.

Initials of authors who received salary or other funding from commercial companies

There were no authors.

URLs to funder websites

https://www.3838.com

Also state whether any sponsors or funders (other than the named authors) played any role in: YES

・Preparing and blinding propolis and placebo tablets

・Decision to publish"

We note that you received funding from a commercial source: Yamada Bee Company, Inc.

Please provide an amended Competing Interests Statement that explicitly states this commercial funder, along with any other relevant declarations relating to employment, consultancy, patents, products in development, marketed products, etc.

**Response: This has been corrected in the cover letter.**

Within this Competing Interests Statement, please confirm that this does not alter your adherence to all PLOS ONE policies on sharing data and materials by including the following statement:

"This does not alter our adherence to PLOS ONE policies on sharing data and materials.” (as detailed online in our guide for authors <http://journals.plos.org/plosone/s/competing-interests>).

**Response: This has been corrected in the cover letter.**

If there are restrictions on sharing of data and/or materials, please state these. Please note that we cannot proceed with consideration of your article until this information has been declared.

**Response: Since there are no particular restrictions on data sharing, we will provide the data set as Supporting Information file.**

Please include your amended Competing Interests Statement within your cover letter. We will change the online submission form on your behalf.

**Response: We have revised the Competing Interests Statement in our cover letter.**

Please know it is PLOS ONE policy for corresponding authors to declare, on behalf of all authors, all potential competing interests for the purposes of transparency. PLOS defines a competing interest as anything that interferes with, or could reasonably be perceived as interfering with, the full and objective presentation, peer review, editorial decision-making, or publication of research or non-research articles submitted to one of the journals. Competing interests can be financial or non-financial, professional, or personal. Competing interests can arise in relationship to an organization or another person. Please follow this link to our website for more details on competing interests: http://journals.plos.org/plosone/s/competing-interests

4. Thank you for stating the following in the Competing Interests section:

"Yoshinari Matsumoto; Grant/research support from: Yamada Research Grant

Kanae Takahashi; Grant/research support/ Speakers bureau: no COI

Kentaro Inui; Grant/research support: Janssen Pharmaceutical K.K., Astellas Pharma Inc., Sanofi K.K., Abbvie GK, Takeda Pharmaceutical Co. Ltd., QOL RD Co. Ltd., Mitsubishi Tanabe Pharma, Ono Pharmaceutical Co. Ltd., Eisai Co., Ltd., Speakers bureau: Daiichi Sankyo Co. Ltd., Mitsubishi Tanabe Pharma, Janssen Pharmaceutical K.K., Astellas Pharma Inc., Takeda Pharmaceutical Co. Ltd., Ono Pharmaceutical Co. Ltd., Abbvie GK, Pfizer Inc., Eisai Co., Ltd., Chugai Pharmaceutical Co., Ltd.,

Tadashi Okano; Grant/research support: AbbVie, Eisai, Mitsubishi Tanabe Pharma Corporation and Nipponkayaku, Speakers bureau: AbbVie, Asahikasei, Astellas Pharma Inc, Ayumi Pharmaceutical, Bristol-Myers Squibb, Chugai Pharmaceutical, Daiich Sankyo, Eisai, Janssen, Lilly, Mitsubishi Tanabe Pharma Corporation, Novartis Pharma, Ono Pharmaceutical, Pfizer, Sanofi, Takeda Pharmaceutical, Teijin Pharma and UCB

Koji Mandai; Speakers bureau: Asahikasei, Eisai

Yutaro Yamada; Speakers bureau: Abbvie, Chugai, Eisai, and Mitsubishi Tanabe

Ayumi Shintani: Grant/research support: no COI, Speakers bureau: Chugai Pharmaceutical Co., Ltd.

Tatsuya Koike; Grant/research support: AbbVie, Astellas Pharma Inc, Bristol-Myers Squibb, Chugai Pharmaceutical, Eisai, Janssen, Lilly, Mitsubishi Tanabe Pharma Corporation, MSD, Ono Pharmaceutical, Pfizer, Roche, Takeda Pharmaceutical, Teijin Pharma, and UCB, Speakers bureau: AbbVie, Astellas Pharma Inc, Bristol-Myers Squibb, Chugai Pharmaceutical, Eisai, Janssen, Lilly, Mitsubishi Tanabe Pharma Corporation, MSD, Ono Pharmaceutical, Pfizer, Roche, Takeda Pharmaceutical, Teijin Pharma, and UCB. This study has not received specific funding from any source.

This research was supported (in part) by Yamada Research Grant."

Please confirm that this does not alter your adherence to all PLOS ONE policies on sharing data and materials, by including the following statement: "This does not alter our adherence to PLOS ONE policies on sharing data and materials.” (as detailed online in our guide for authors http://journals.plos.org/plosone/s/competing-interests). If there are restrictions on sharing of data and/or materials, please state these. Please note that we cannot proceed with consideration of your article until this information has been declared.

Please include your updated Competing Interests statement in your cover letter; we will change the online submission form on your behalf.

Please know it is PLOS ONE policy for corresponding authors to declare, on behalf of all authors, all potential competing interests for the purposes of transparency. PLOS defines a competing interest as anything that interferes with, or could reasonably be perceived as interfering with, the full and objective presentation, peer review, editorial decision-making, or publication of research or non-research articles submitted to one of the journals. Competing interests can be financial or non-financial, professional, or personal. Competing interests can arise in relationship to an organization or another person. Please follow this link to our website for more details on competing interests: http://journals.plos.org/plosone/s/competing-interests

**Response: This has been corrected in the cover letter.**

5. We note that you have indicated that data from this study are available upon request. PLOS only allows data to be available upon request if there are legal or ethical restrictions on sharing data publicly. For information on unacceptable data access restrictions, please see http://journals.plos.org/plosone/s/data-availability#loc-unacceptable-data-access-restrictions.

In your revised cover letter, please address the following prompts:

a) If there are ethical or legal restrictions on sharing a de-identified data set, please explain them in detail (e.g., data contain potentially identifying or sensitive patient information) and who has imposed them (e.g., an ethics committee). Please also provide contact information for a data access committee, ethics committee, or other institutional body to which data requests may be sent.

b) If there are no restrictions, please upload the minimal anonymized data set necessary to replicate your study findings as either Supporting Information files or to a stable, public repository and provide us with the relevant URLs, DOIs, or accession numbers. Please see http://www.bmj.com/content/340/bmj.c181.long for guidelines on how to de-identify and prepare clinical data for publication. For a list of acceptable repositories, please see http://journals.plos.org/plosone/s/data-availability#loc-recommended-repositories.

We will update your Data Availability statement on your behalf to reflect the information you provide.

**Response: Since there are no restrictions, we will upload the data set as a supporting information file.**

Reviewer #1: Dear authors,

Thank you for this well written and thoroughly designed study on the effect of propolis on disease activity in RA. i have to admit I did not know this substance, so first of all thank you for this. Although this is a negative trial, eg the effect is missing, I believe this study to be sound and deserves publication. However, before this is warranted, a few questions should be answered

- In this RCT only women were included. Why?

**Response: Eighty percent of rheumatism patients are women, and the main reason was that we thought it might be easy to recruit cases. In addition, there are often differences in disease activity between men and women, and the number of cases was small, so we limited it to women to avoid the effects of gender. In the future, I think it might be necessary to conduct research on men.**

- Moderate disease activity is perhaps not an ideal state to discover very small effects in inflammation changes. One could also think of inhibition of flares, or as adjuvans in early RA with intensive treatment strategies. However, doing this in practice is perhaps a lost effort as this trial clearly shows no benefit at all of the supplements.

- For the power calculation, there is an effect size of 0.5 assumed. Perhaps this was too ambitious and more persons were needed to detect very small effect sizes... I would write such a statement in limitations.

**Response: That's exactly what you say. This point has already been mentioned in the discussion as the first limitation. We added a statement about the possibility of different results if different targets are set and similar tests are performed. The effect size of 0.5 is an estimated value from the dietary intervention study mentioned in the paper. The EULAR also defines a “clinical response” as an improvement in DAS of 0.6 or more for moderate disease activity. We adopted 0.5, which is smaller than 0.6 and is the expected effect from the dietary intervention study. An intervention trial with more patients may be necessary. The overestimate of the anti-inflammatory effect of propolis has been added to limitation.**

- I now have mastered all knowledge on propolis via Wikipedia and have read that propolis is very heterogeneous in content. Could it be that one specific type of propolis does work better than the other? Why was the Brazilian propolis chosen?

**Response: Propolis has a different composition depending on the place of origin (PMCID: PMC6864204). Therefore, the percentage of cinnamic acid, which is expected to have an anti-inflammatory effect, for example, may vary depending on the place of origin, which may result in some effects as you mentioned. However, since there is no report that verifies the clinical effects of propolis according to the place of origin, it is only an estimation.**

**We selected Brazilian propolis because its anti-disease activity effect was verified in a mouse model of rheumatoid arthritis (PMID: 21861090, i.e. reference 10).**

Good luck!

Reviewer #2: A two-arm randomized placebo-controlled clinical trial (n=80) was conducted to compare the effects of Brazilian propolis to placebo on change in disease activity in RA patients. No significant differences were observed in Disease Activity Score or any secondary endpoints between the two arms.

Minor revisions:

1- Line 226: Consider editing the phrase “If no normality was observed...”

**Response: We have changed the text in the revised version accordingly.**

2- Line 230: Indicate the underlying covariance structure used in the mixed-effects models and the criteria for selecting it.

**Response: Because we did not want to make any assumption of the covariance structure, we a priori determined to use unstructured covariance matrices. In the manuscript, we now added “The covariance structure within patients in the model was unstructured.” in the revised version.**

3- Line 252: Provide a measure of dispersion (range or interquartile range) for the median age.

**Response: We have added the content on revised version. The median and interquartile range of age of the overall study cohort was 61.5 (56.0, 67.3) years.**

4- The abstract indicates that analysis of covariance methods were used to analyze the data; the statistical analysis section, however, states that mixed-effects models were used. Please clarify. The statistical analysis section should include a comprehensive listing and explanation of all the statistical methods used for the analysis.

**Response: We are sorry for making a confusing description. The analysis of covariance was main analysis. So, the description in abstract was correct. We changed "statistical analysis" in revised version to avoid misunderstanding. The description of secondary analysis has also been revised.**

5- The study is likely underpowered to test the interaction of arm by time. Plus the sample size justification/power calculation is based upon a t-test, not a test of the interaction effect. Please edit the manuscript for clarification. If tests of interaction effects were conducted, the results have not been presented. If tests of interactions are conducted indicate if the following procedure was implemented when testing interaction effects and main effects. If the interaction effect is significant, provide an interpretation of the results, but do not test main effects because the tests for main effects are uninteresting in light of significant interactions. If interaction effects are non-significant, drop the interaction effects from the model and test the main effects. Determining which results to present when testing interactions is often a multi-step process.

**Response: Because the main analysis was conducted with the analysis of covariance instead of using mixed effect model with time-treatment interaction, we did not consider the interaction effect in the sample size justification. The mixed-effects model analyses were performed for the primary endpoints to confirm the robustness of results on the LOCF completion of missing values. In the mixed effect model, we included the interaction terms between time (as categorical variable) and treatment variables in order to estimate the effect of the treatment at 24 weeks. We did not intend to test for the interaction (i.e., to test whether the treatment effect varies overtime). We do not recommend dropping the interaction term from the model because the dropping the interaction term will result in biased estimate in the 24 week treatment effect.**

6- When presenting results from mixed effect models, please clarify what, if any, other variables than those listed in the tables were included in the model.

**Response: The results in Fig. 3 and Fig. 4 are analysis of covariance, so the statistics have been corrected.**

7- Indicate the date range subjects were enrolled in the study.

**Response: We have included what you pointed out in revised version.**

Reviewer #3:

Line 39: please elaborate on the term moderate disease activity, was that assessed by use of DAS28-CRP, or?

**Response: The disease activity in this part is for DAS28-ESR. We have corrected the content of the text.**

Line 42: the abstract describes the use of propolis tablets containing 508.5 mg of propolis whereas the trial registration describes the use of tablets containing 378 mg propolis, please clarify (https://rctportal.niph.go.jp/en/detail?trial_id=jRCTs051180142)

**Response: Thank you for your very meaningful suggestions. We were completely unaware of the contradiction you pointed out. The propolis dose in the manuscript was correct, and the dose registered in jRCT was incorrect. It is presumed that there was some calculation error when registering with jRCT. We have already submitted an amendment application to the Japanese Ministry of Health, Labor and Welfare regarding the description of jRCT. Thank you so much.**

Line 43: were the tablets similar in shape and color?

**Response: Both propolis tablets and placebo tablets had the same tablet form. Both the propolis and placebo tablets were coated in white so that they were indistinguishable in their looks.**

Line 204: I would recommend including an analysis of the ITT population, defined as every patient randomized as that would minimize a potential bias due to exclusion of patients. Moreover, the ITT analysis reflects the practical clinical scenario because it admits noncompliance and protocol deviations, pls see https://www.ncbi.nlm.nih.gov/pmc/articles/PMC3159210/.

**Response: Thank you for pointing this out. As shown in the flowchart in Figure 1, all 80 randomized cases were included in the FAS. ITT and FAS populations matched.**

Line 207: I would recommend adapting the FAS population to all patients randomly assigned to a treatment group having at least one efficacy assessment after randomization and participants who never consumed the test food.

As highlighted by EMA (https://www.ema.europa.eu/en/documents/scientific-guideline/ich-e-9-statistical-principles-clinical-trials-step-5_en.pdf) there a only very few circumstances that may lead to the exclusion of randomized subjects in an FAS analysis (one circumstance already being mentioned in the manuscript, “participants who never consumed the test food”).

If the criteria “participants found to be ineligible after randomization” is to be used this paragraph must be expanded (pls see link above).

**Response: We absolutely agree with you. We removed the phrase "participants found to be ineligible after randomization" from the definition of FAS in the manuscript and added it to the definition of PPS. Since there were no cases that met the criteria for "participants found to be ineligible after randomization," the results of the analysis remain unchanged.**

Line 218: I would recommend defining the Safety Analysis Set as all randomized patients with at least one safety assessment after randomization.

**Response: Thank you for your suggestion. We have also changed the description of the SAS definition accordingly.**

Line 230: why include week 12 data in the analysis of the primary endpoint?

**Response: This is because the EULAR algorithm for the treatment of patients with rheumatoid arthritis by medication is focused on the evaluation of changes in disease activity at 3 and 6 months after the start of treatment (reference 4).**

Table 1: did none of the patients receive csDMARDS of ts-DMARDs?

**Response: The number of csDMARDs other than MTX users has been added to Table 1. In the first version of Table 1, bDMARDs users included ts-DMARD users. Therefore, in the revise version Table 1, the type of medication used was changed from bDMARDs user to b-/ts-DMARD user. There were 5 ts-DMARD users out of 80 participants in the study, including Group C (n = 1, 2.5%) and Group P (n = 4, 10.0%).**

Figure 1: needs to be updated to include i.e. reasons for discontinued intervention (box-row 5 from the top, pls see https://www.equator-network.org/reporting-guidelines/consort/)

**Response: Thank you for pointing this out. We have added the reason for study cancellation in 8 participants to Fig 1.**
