# Supplementary material for: Double-blinded randomized controlled trial to reveal the effects of Brazilian propolis intake on rheumatoid arthritis disease activity index; BeeDAI
Source: PLoS One. 2021 May 27;16(5):e0252357. doi: 10.1371/journal.pone.0252357 (PMC8158978; doi:10.1371/journal.pone.0252357)
Supplement: Attachment — Submitted filename: Response to Reviewers.docx [file pone.0252357.s010.docx]

Dear Editor and Reviewers;

We would like to thank you for your very constructive criticisms and editorial suggestions regarding our manuscript. These were very helpful in our sincere efforts to improve our manuscript. We addressed all the concerns point-by-point, **depicting Reponses by use of bold font**, and revised the manuscript accordingly. We hope that our response meets with your approval. We indicate revised wording with yellow highlighting in the text.

Reviewer #1: Dear authors,

Please put in limitations that

1. These results only apply to women, and that studies in men should be considered.

**Response: Thank you for your comment. We have added a note about the need for studies with men. We added a sentence in the text, “Third, this study was conducted only on women. It would be desirable to verify the effect of propolis on male patients with RA in the future.**”

2. Refrase line 324-326: "We might have overestimated the effectiveness of propolis and might need to develop clinical studies to verify less efficacy in the future." You dont want to verify less efficacy, you want to make sure that propolis has or do not has an effect. Therefore, you need to have an improved power calculation based on more realistic effect sizes in a representative population.

**Response: Thank you for your comment. We have corrected the description in this section. We changed the sentence to “We need to have an improved power calculation based on more realistic effect sizes in a representative population.”.**

Reviewer #3: Thanks for your replies to the comments provided

As previously pointed out I would strongly urge you to adapt the description of your FAS population according to ICH as there are a limited number of circumstances that might lead to excluding randomized subjects from the full analysis set including

- the failure to satisfy major entry criteria (eligibility violations),

- the failure to take at least one dose of trial medication and

- the lack of any data post randomisation

**Response: Thank you for your advice. Actually, no participant violated the above conditions, but we added 2 items to the definition part of FAS as 4) and 5). Participants who have never taken the test tablet have already been mentioned.**

The trial figure is to be updated according to the CONSORT guideline (https://www.equator-network.org/reporting-guidelines/consort/)

**Response: Thank you for pointing this out, we have modified the flowchart in Fig.1 to match the CONSORT diagram.**

The manuscript describes that in Group C the study was discontinued in 4 cases, this is not reflected in figure 1

**Response: Thank you for pointing out our misleading statement. No one in the control group failed to complete the test. It was correct that the number of discontinued intervention is zero. At the final check stage, four subjects took less than 70% of test tablets. The description in the text and Figure 1 have been revised to make it easier to understand.**
